# Understanding factors associated with attending secondary school in Tanzania using household survey data

**DOI:** 10.1371/journal.pone.0263734

**Published:** 2022-02-25

**Authors:** Carla Pezzulo, Victor A. Alegana, Andrew Christensen, Omar Bakari, Andrew J. Tatem

**Affiliations:** 1 WorldPop, School of Geography and Environmental Science, University of Southampton, Southampton, United Kingdom; 2 Population Health Unit, Kenya Medical Research Institute—Wellcome Trust Research Programme, Nairobi, Kenya; 3 Centre for Tropical Medicine & Global Health, Nuffield Department of Medicine, University of Oxford, John Radcliffe Hospital, Headington, Oxford, United Kingdom; 4 Plan Denmark (PlanBørnefonden), Copenhagen, DK, United Kingdom; 5 Tanzania DataLab (dLab), Dar es Salaam, Tanzania; University of Salamanca, SPAIN

## Abstract

**Background:**

Sustainable Development Goal (SDG) 4 aims to ensure inclusive and equitable access for all by 2030, leaving no one behind. One indicator selected to measure progress towards achievement is the participation rate of youth in education (SDG 4.3.1). Here we aim to understand drivers of school attendance using one country in East Africa as an example.

**Methods:**

Nationally representative household survey data (2015–16 Tanzania Demographic and Health Survey) were used to explore individual, household and contextual factors associated with secondary school attendance in Tanzania. These included, age, head of household’s levels of education, gender, household wealth index and total number of children under five. Contextual factors such as average pupil to qualified teacher ratio and geographic access to school were also tested at cluster level. A two-level random intercept logistic regression model was used in exploring association of these factors with attendance in a multi-level framework.

**Results:**

Age of household head, educational attainments of either of the head of the household or parent, child characteristics such as gender, were important predictors of secondary school attendance. Being in a richer household and with fewer siblings of lower age (under the age of 5) were associated with increased odds of attendance (OR = 0.91, CI 95%: 0.86; 0.96). Contextual factors were less likely to be associated with secondary school attendance.

**Conclusions:**

Individual and household level factors are likely to impact secondary school attendance rates more compared to contextual factors, suggesting an increased focus of interventions at these levels is needed. Future studies should explore the impact of interventions targeting these levels. Policies should ideally promote gender equality in accessing secondary school as well as support those families where the dependency ratio is high. Strategies to reduce poverty will also increase the likelihood of attending school.

## Introduction

Sustainable Development Goal (SDG) 4 aims to ensure inclusive and equitable quality education for all by 2030, and at leaving no one behind, through targeting at equal access to any level of education, increasing the participation rate of youth in education (SDG 4.3.1) and reducing out-of-school rates (SDG 4.1.4) [1]. Trends at the global level showed that nearly one-fifth of the global population among adolescents (lower secondary school age) and youth (upper secondary school age) are out of school, and that progress towards reducing numbers of adolescents and youth who are out-of-school has stagnated in LMICs [2], Moreover, according to UNESCO, only a reduced proportion of those who complete primary school will complete secondary school [3], and this pattern is seen in particular among the most vulnerable populations.

Benefits from education such as economic growth, poverty reduction and decreasing inequalities are well established [4, 5]. Associations between school attendance and positive health outcomes and choices have been explored in different contexts [6, 7]. Socio-economic and demographic household characteristics such as wealth and parents’ educational attainment have been identified as influencing child and youth education [8], and can generate a barrier to access or remaining in school. Geographic variations and disparities in accessing schools are also present within countries and across groups, with contextual characteristics such as distance to school and number of teachers, among others, playing a major role in rates of enrolment to primary schools [8]. Sub-Saharan Africa is the region presenting the highest rates of out-of-school in both primary and secondary education, with 97.5 million children (generally defined by the United Nations as ranging between the age of 10 to 15), adolescents (10 to 19) and youth (15 to 24) being out of primary and secondary school [9, 10].

Adolescence and youth are widely recognised as being critical ages for rapid development and for establishing the foundation for future health [11] and to expand life opportunities, where education is crucial for their growth and development towards adulthood. Accessing and attending school play a role as a protective factor against poverty, child marriage and early pregnancies, among other risks, leading to positive repercussions on an individual’s health and development, on a country’s economy and the wellbeing of a society [12, 13]. Moreover, there has been a general call to join the effort to gather better data on the youth not in employment, education or training (NEET) rate, where the risk of falling within the NEET category is generally associated with low educational attainment and poor literacy and numeracy [2].

Recent evidence suggests that countries in Sub-Saharan Africa are most affected [14]. For example, Tanzania showed great achievements in enrolment for basic education, however the out-of-school rates are still very high, with 6.7 million among children, adolescents and youth of primary and secondary school age being out of school (UIS UNESCO data for 2016) [13]. This is particularly the case in remote rural areas, with girls lagging behind boys in the transition from primary to secondary education, and a girls-to-boys ratio in upper secondary and higher education of about 1:2 [15].

### Education policies and trends of schooling over time in Tanzania

With one of the world’s largest young populations (43% aged 0–14 and 32.5% aged 10–24 in 2012, [16]), Tanzania has put education as a national priority since its independence in 1961. All Tanzanian governments have supported emerging movements emphasising the need for free primary education and prompted investment strategies, moved by the Country’s aspiration to become a middle-income country by 2025 [17–20]. If post-independence education strategies saw an increment in enrolment rates [21], with the 1980s and the implementation of structural adjustment programs there has been a reduction of public expenditure on education leading to a decrease in rate of primary and secondary school participation, particularly among the most vulnerable [17, 18, 20]. In 2000, like other African countries, Tanzania implemented a universal primary education (UPE) policy, which increased both primary enrolment rates, as well as the demand for secondary education. Policy documents emphasising the intention to increase secondary school enrolment by adding classrooms to existing schools, as well as building new regional schools followed, and strategies to ensure that each ward in the country had at least one secondary school were set in 2008 [19, 20, 22, 23]. In December 2015, Tanzania’s new government took a further step: it abolished all school fees previously required to enter lower-secondary schools in the country, establishing fee free basic education for all. The five-year Education Sector Development Plan (ESDP) just announced, was established to achieve key targets and indicators for education and capacity development in the context of the Sustainable Development Goal 4, which requires States to ensure that everyone “completes free, equitable and quality primary and secondary education” [19].

Secondary school net attendance rates (NARs) in Tanzania have seen an increment over the past decades, raising from as low as 7.3% in 2004–5 to 23% in 2015–16 (Measure DHS) nationally [24]. Wide regional inequalities in attendance rates persist, with mainland Tanzania regions like Kilimanjaro, Iringa and Dar Es Salaam leading with NARs ranging between 40% and 60% in 2015–16 and rising from NARs ranging between 14% and 20% in 2004–5. Regions like Rukwa, Tabora, Katavi and Shinyanga, have fallen behind, with NARs for secondary being between 10 and 14% in 2015–6, rising from NARs which ranged between 0.3% and 2.6% in 2004–5 [24]. Looking at trends for all regions in Mainland Tanzania enables improved understanding of within-country disparities and provides a framework for policies and measures implemented over the past years to increase secondary enrolment and attendance.

### Factors influencing schooling and barriers to access

Following the increase in enrolment and attendance rates in primary and lower secondary schools, challenges emerged in meeting adequate resources and infrastructure needs and in providing a sufficient number of personnel to deliver educational programs and quality education [17, 22, 25]. Barriers to access and school attendance still persist in low and middle-income countries, and a wide range of literature has explored drivers of school access and attendance through both observational and experimental studies, such as through randomised evaluations. While observational studies aimed at looking at factors associated with access and attendance, randomised evaluations allowed disentangling the causal impact of programs designed to improve access and attendance [26]. Among all factors explored, location and distance to school, costs, age of the child, parents’ background and household characteristics were found to be significantly associated with schooling in Tanzania [21] as well as in other contexts [8]. There is wide evidence that distance to school and enrolment to primary and secondary school are often statistically significantly related [27], however the magnitude of the relationship has also been found to be small [28]. Quality of school provisions, including the pupil to teacher ratio, were also found to be associated with school attendance [29–31], although the correlation was not significant in all contexts [21]. Overall, in their work, Burke and Beegle (2004) concluded that policies aiming at increasing attendance should focus more on the household’s demand for schooling (e.g., reducing costs) than on the supply of education services (school infrastructures and quality). This was also supported by findings from Dreibelbis et al., 2013 [32], who concluded that household characteristics, such as socio-economic status and demographic features like gender were more important predictors of absence than water and sanitary conditions at school level in Kenya. Particularly among economists there is a consensus on the need for schooling to be cost-beneficial to the household [26], however, the relationship between child labour and attendance is not always clear [30].

Similar to findings from observational studies, randomised evaluations looking at the impact of programs that aimed to increase access and attendance and improve learning outcomes in low-and middle-income countries found that reducing prices, making schools more cost-effective, assigning merit scholarships and distance to schools all have an impact on increasing school participation [26, 27, 33]. Moreover, it was found that programs that improved knowledge on returns and awareness on costs-benefits from attending school [27] and that provided information on how earnings rise with education can increase schooling even more cost-effectively [26]. A wide range of literature from behavioural and development economics also looked at the effects of conditional cash transfers (CCT), which usually provide financial support to poor mothers if their children obtain basic medical care and attend school regularly, on actual school participation [27, 33–35]. Questions related to factors driving school attendance in low- and middle-income countries have been increasingly gaining more policy makers’ attention as governments have begun to commit to free basic education. Nevertheless, the existing literature on education in developing countries remains overly focused on trends at primary education level. Here, the aim of our study was to improve understanding on the drivers of adolescents and youth school attendance versus non-attendance for the secondary school age group in Tanzania (14 to 19 years old), where secondary school trends of enrolment and attendance have experienced a peculiar path since the Country’s independence [19, 23]. Moreover, this study aims to contribute to the discussion around the relationship between school attendance and contextual factors, in particular exploring distance to school and pupil to qualified teacher ratio using geographical information system (GIS)-based data sources and spatial analysis, which are novel to such area of study.

A two-level (multilevel) random intercept logistic regression analysis was used to estimate the probability of attending secondary school, including children of adolescents or youth-level, parents and household level variables using the 2015–16 Tanzania Demographic and Health Surveys (DHS). Moreover, contextual variables such as travel time to the nearest secondary school and pupil to qualified teacher ratio were also explored in the analysis to test whether they also contribute to the probability of attending school. The use of a multilevel modelling framework allowed for accounting for the multistage sampling design of the survey data and for the hierarchical structure of the data, where individuals are nested within households and clusters [36, 37]. Such multilevel modelling techniques have been used previously in the literature with Demographic and Health Surveys (DHS) [38]. The multilevel analysis also allowed for testing contextual variables as factors associated with the outcome variable of school attendance, still accounting for intra-cluster correlation and data hierarchy [37, 39–41]. Finally, this research focuses on school attendance rather than enrolment, since a child may be enrolled at the beginning of the year but may not be attending school [21].

## Methods

### Data

We used the Demographic and Health Surveys (DHS) data for Tanzania (2015–16 DHS, n = 595 clusters) [24, 42] downloaded from the MEASURE DHS website, and extracted a potential list of key individual (age and sex of the child, education attainment of the head of the household and parents) and household (household wealth index, number of children under the age of 5 in the household) level characteristics used as variables (or factors) that impact secondary school attendance. The DHS program collects nationally representative household surveys in over 90 countries, which provide indicators on a wide range of topics including population, wealth, maternal and child health, fertility and family planning, nutrition, and education indicators. DHS sampling design is implemented using a two (or sometimes three) stages stratified sampling design using censuses as sampling frames. During the first stage of selection, enumeration areas (EAs), also known as clusters, are selected by using a probability proportional-to-size selection (EA size). During the second stage household are usually sampled from a complete household listing in the selected EAs using systematic sampling. Specific details on the sampling procedures for can be found on the DHS final report [24] and DHS Sampling Manual [42]. Clusters (also used interchangeably with primary sampling units (PSU) and enumeration areas (EAs)) are defined as a group of households in the same area or a block (if in urban areas) selected for the interview within the complex survey design used by the DHS.

The adjusted net attendance rate used as outcome variable in this study was defined as the total number of students of the official secondary school age-group attending primary or secondary or higher education at a reference academic year, following indications from UIS UNESCO and the DHS methods [43, 44]. It therefore included children of official school age who accessed school earlier or later than the normal enrolment age and was expressed as a percentage of the corresponding population [45], giving a more precise picture of participation to school. The designated age-ranges for secondary school in Tanzania ranges between 14 to 19 years old. The numerator was the de facto total population of secondary school age attending secondary school (or primary, secondary or higher in the case of the adjusted rate) while the denominator was the total number of de facto secondary school age population. The age at the start of the academic year was used to determine the eligible secondary school age population used in the numerators and denominators for the net attendance rate [44]. To establish these age ranges, full information on the date of birth of the child in question was triangulated with the start of academic year, to account for temporal gap between the interviews and the start of the academic year. The out-of-school rate for secondary school was calculated by subtracting the adjusted net attendance rate for secondary education from 100%.

### Cluster level contextual variables

Alongside individual and household level factors, contextual level factors such as travel time to nearest secondary school (a proxy for access to school) and pupil to teacher qualified ratio (PQTR, a proxy for school service offered / quality) [46] were constructed. Cluster level information about travel time to the nearest secondary school were extracted from the gridded estimates to account for travel time as contextual variable. The average travel time to nearest secondary school were extracted at each cluster. The methodology for computing travel time has been documented in previous studies [47–50]. Firstly, school locations were triangulated with ancillary spatial data on elevation (DEM), obtained from HydroSHEDS dataset [51], land cover, obtained from MERIS GlobCover [52], and road networks, assembled from Open Street Maps (OSM) and other online resources such as the National Geospatial-Intelligence Agency (NGA) [53] and MapCruzin [54], using Access Mod version 5 software [55]. Secondly, a raster surface of travel times to the school locations that include walking across land cover and motorised travel along major roads was generated and used in the analysis.

The focal statistics tool available under ArcGIS Spatial Analyst (ESRI ArcGIS 10.7) was employed to calculate means within a 2km or 5km buffer around each cluster, depending on whether they were an urban or rural cluster in order to take into account the urban/rural split in survey sampling, and the respective 2km and 5km displacement of DHS clusters.

The PQTR is defined as the average number of pupils per qualified teacher at a given level of education, based on headcounts of both pupils and teachers [44], and it was also tested as a contextual variable. The PQTR gives indication of how many teachers per pupil are present in a school, and therefore how much care and attention can be given to each individual pupil. A qualified teacher is one who has at least the minimum academic qualifications required for teaching his/her subjects at the relevant level in a given country [44]. Information on pupil-to-qualified teacher ratio (PQTR) was extracted from online education database for Tanzania (www.africaopendata.org [46]). This included information on the number of children enrolled at each school and teachers in every classroom of each secondary school. The higher the pupil-qualified teacher ratio, the lower the relative access of pupils to qualified teachers, where a high pupil-teacher ratio suggests that each teacher is responsible for a large number of pupils. On the contrary, it is generally assumed that a low pupil-qualified teacher ratio signifies smaller classes, which enables the teacher to pay more attention to individual students [44]. A GIS inverse distance weighting (IDW) interpolation technique was used to create a continuous surface of PQTR in Tanzania. S1 Fig and S1 Text show the distribution of the pupil-qualified teacher ratio (PQTR) in Tanzania for each school and a relative surface. PQTR values for each DHS cluster were extracted using focal statistics around urban areas (using a buffer of 2km) and rural areas (5km). Information about children of secondary school age were linked to the PQTR quality indicator, and values at cluster level were therefore employed in the modelling framework as a contextual variable to understand factors associated with access to secondary school.

### Analysis of the drivers of secondary school attendance

Based on the data availability and on the theoretical relationship discussed in previous studies between determinants and school attendance [among others: 8, 21], a full list of individual, family and household and contextual characteristics was derived and their association with the outcome variable tested using bivariate analysis and a forward stepwise covariate selection process.

A bivariate analysis was performed to identify demographic and socio-economic characteristics associated with the adjusted secondary school attendance ratio. This descriptive analysis tested for differences within groups using F test and t-test for equality of means (for continuous variables) adjusting for sample design and with a significance level of p<0.05. Data were analysed with Stata/SE 16.0 for Windows [56] and adjusted for the survey sampling design.

Additionally, a forward-stepwise covariate selection procedure using an alpha level of 0.05 was implemented to identify a parsimonious set, while collinearity between independent variables was explored using VIF statistic. Collinearity was considered high for covariates with a VIF greater than 4, which indicates a twofold increase in the standard error of a regression coefficient, in presence of collinearity. In case of two collinear variables (with high VIF), the variables with the highest R^2^ statistic when compared to the outcome variable was retained. Interaction terms for variables age and level of education of the household head were initially tested outside the modelling stage, followed by an assessment within the modelling stage, where they resulted to be not significant, and therefore not included in the final model.

A two-level (multilevel) random intercept logistic regression analysis for the probability of attending secondary school was conducted, with individuals nested within primary sampling units (clusters) [39, 40], and the notation for a two-level random intercept model for binary responses as follows:

$$log\;(\frac{\pi_{ij}}{{1-\pi }_{ij}})=\beta_{0}+\beta_{1}x_{1ij}+\beta_{2}x_{2j}+u_{j}$$ (1)

where, *u_j_*~N(0, $\sigma_{u}^{2}$), and *π_ij_* = is the probability of an event occurring for the *i* level 1 unit in the *j* level 2 unit; *β*_0_ is the log-odds that *y* = 1 when *x* = 0 and *u* = 0; *β*_1_ is effect on log-odds of 1-unit increase in *x* for individuals in same group; *u_j_* is the effect of being in group j on the log-odds that *y* = 1; also known as a level 2 residual; $\sigma_{u}^{2}$ is the level 2 (residual) variance, or the between-group variance in the log-odds that *y* = 1 after accounting for *x*; *x*_1*ij*_ is a generic level one nested within level 2 independent variable; *x*_2*j*_ indicates a level two independent variable. The response variable “School attendance” was binary distributed, with value equal to 0 when the eligible children of secondary school age wasn’t attending school, and value equal to 1 when the eligible children of secondary school age was attending school. The analysis aimed at describing factors associated with children attending school. At the first level, we defined the child, parents or household level; with level two we defined the cluster (community/contextual) level. Due to the sample size, there was no rationale for having either parents or household as second level in the model. Interaction terms were also explored outside the modelling frameworks and tested within the full multilevel models to assess their significance level. Log-likelihood tests for goodness of fit were performed between a simple logistic regression and a null model with random intercept at level two. Adding a random intercept at cluster level proved to be statistically significant and therefore random intercepts were retained. Finally, to find the best possible full multilevel model, log-likelihood tests for goodness of fit were also performed by comparing the null model with random intercept and by adding one independent variable at the time.

For ease of interpretability, our results for the multilevel models are presented using odds ratios, taking the exponent of the log-odds and confidence intervals at 95% of probability. Intraclass correlation coefficients (ICC) were calculated for the final model. ICC measure the correlation of the observations of the children belonging to the same cluster (community), and it is defined as the variance between clusters divided by the total variance, where the total variance is formed by the variance between groups and the variance within groups [41]. Finally, adjusted mean predictions for the fixed portion of the model were calculated after running the multilevel logistic model, to compute the probability of accessing secondary school for selected characteristics in the model, holding all the other independent variables in the model at their mean values.

### Ethical approval

University of Southampton number: 45660.

## Results

### Factors associated with secondary school attendance

**Fig 1:**
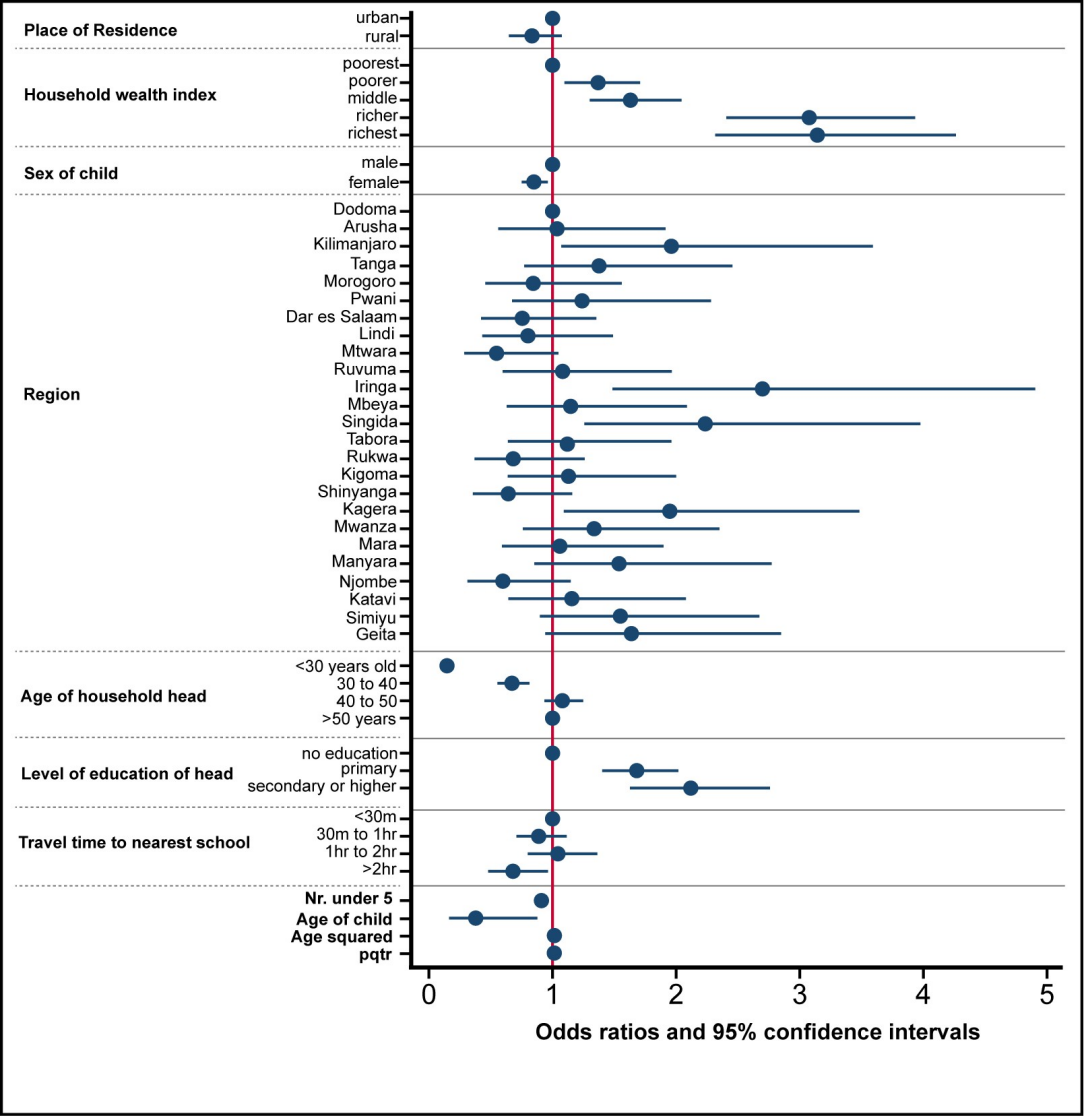
Plotted coefficients for fixed effect covariates from a multilevel-multivariate analysis of drivers of school attendance for Tanzania. Odds ratios and 95% confidence intervals from the two-level logistic regression model of school attendance among children of adolescents or youth-level using DHS data in Tanzania (2015–16 DHS, N = 6,197). Fixed effects only were plotted. A map of the regions of Tanzania is presented in S2 Fig.

Results from the two-level logistic regression model suggested that individual and household characteristics played a major role in explaining the difference in children’s attendance to secondary school, while community characteristics such as travel time to the nearer secondary school and pupil to qualified teacher ratio, only explained a small share of the variance in school attendance. Fig 1 shows plotted coefficients for fixed effects covariates from the two-level logistic regression model, and S5 Table in the Supplements presents odds ratios and 95% confidence intervals for secondary school attendance for the same model. S1–S3 Tables show respectively Tanzania 2015–16 DHS definitions of each variable, summary statistics for selected socio-demographic and background characteristics of children of secondary school age, and results for a bivariate analysis of various indicators by school attendance status in Tanzania, which was used to determine the inclusion of variables in the final two-level logistic regression model. S5 Table in the Supplements presents odds ratios and 95% confidence intervals for secondary school attendance for the same model, and S4 Table presents additional statistics.

**Fig 2:**
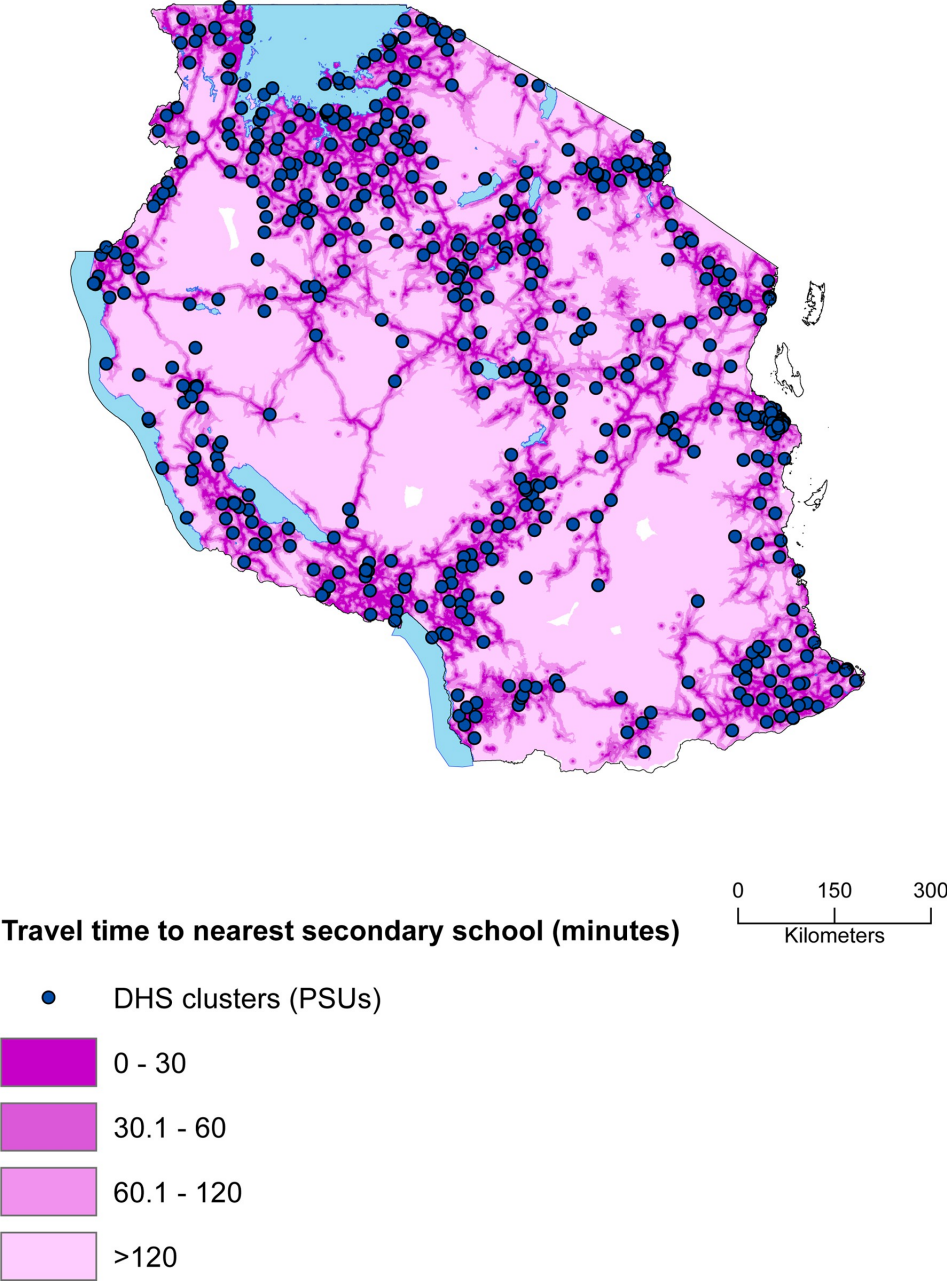
Geographic variation in theoretical travel time to the nearest secondary school. The blue dots are locations of DHS clusters (n = 595); Light blue areas are large water bodies extracted from the open source repository DivaGis (www.diva-gis.org). DHS clusters, also called primary sampling units (PSUs) are typically census enumeration areas (EAs) which form the survey cluster.

Specifically, being in the richer and richest wealth quintiles increased odds of attending secondary school by at least two times (OR: 3.01, CI = 2.41,3.93 the former; OR: 3.14, CI = 2.32,4.26 the latter), as compared to being on the poorest quintile. Belonging to a household with household heads who attained primary, secondary or higher school levels, compared to those where heads had no education increased the odds of accessing school by 68%, the former (OR: 1.68, CI = 1.4, 2.02), and two times the odds of attending school, the latter (OR: 2.12 CI = 1.63, 2.76). For older household heads, the odds of attending school are greater. The same patterns were also shown in the model including mothers and father’s information on education, and removing information about household heads, where children with parents having higher levels of education were more likely to attend secondary school (S6 and S7 Tables). Other household level characteristics were associated with attending school in the household level model (S5 Table and Fig 1), such as the number of children under the age of 5 present in the household. The higher the number of children under 5, the less likely the secondary school age child was to access school, by 10% for each additional child. An older age of the child was associated with decreased odds of attending secondary school, in particular for every one year increase in age, the odds of attending was reduced by 60% (0.38, CI = 0.16, 0.88). Regional level variations were also present; compared to Dodoma region, children residing in Kilimanjaro, Iringa, Singida and Kagera were more likely to attend secondary school (S2 Fig). Travel time to the nearest school only seemed to be partially associated with school attendance. Those living within 2 hours from the nearest school (about 91% of the sampled population) do not present significant variation in school attendance, meaning that distance does not matter. Only when looking at the small portion of children residing in areas further away from secondary schools (9% of sampled population living above 2 hours far from a secondary school), distance was a barrier to access, as they were 30% less likely to attend school, compared to those who are within 30 minutes distance from the nearest school. Fig 2 presents cluster locations superimposed to the travel time to nearest secondary school map in Tanzania, derived as a combination of walking across land cover and motorised travel along major roads [47]. The map shows that 91% of clusters fell within 2 hours travel time to the nearest secondary school, 43% of clusters were within 30 minutes, 30% between 30 minutes and 1 hour and 18% between 1 to 2 hours. Only 9% of clusters, were greater than 2 hours from nearest school.

The PQTR showed some significance in explaining school attendance status, although its effect was almost null (OR: 1.01, CI = 1,1.03), where an odds ratio equal to one indicates no change in the odds after a change of one unit in the predictor. The cluster level random intercepts were reduced as compared to the null models (estimate for null model = 0.644, CI 95%: 0.521; 0.794), but it was still significant, meaning that part of the cluster level variability remains unexplained. Examining the intraclass correlation for Tanzania, only 8% of the variation in school attendance lay between clusters, while 92% lay within clusters between children. This suggested that the majority of the variation in access to secondary school attendance was due to individual and household level factors, more than on contextual (cluster) factors. This was also shown in the caterpillar plots (S4 Fig), where estimated residuals for all clusters in the sample were plotted with confidence intervals. For almost all clusters, the 95% confidence interval overlap the horizontal line at zero, and only a few are above or below average. This indicated that only a small part of the variation in school attendance was due to cluster level factors, while the majority of the variance was actually explained at the individual and household level.

### Predicting the probability of accessing secondary school for selected characteristics

**Fig 3:**
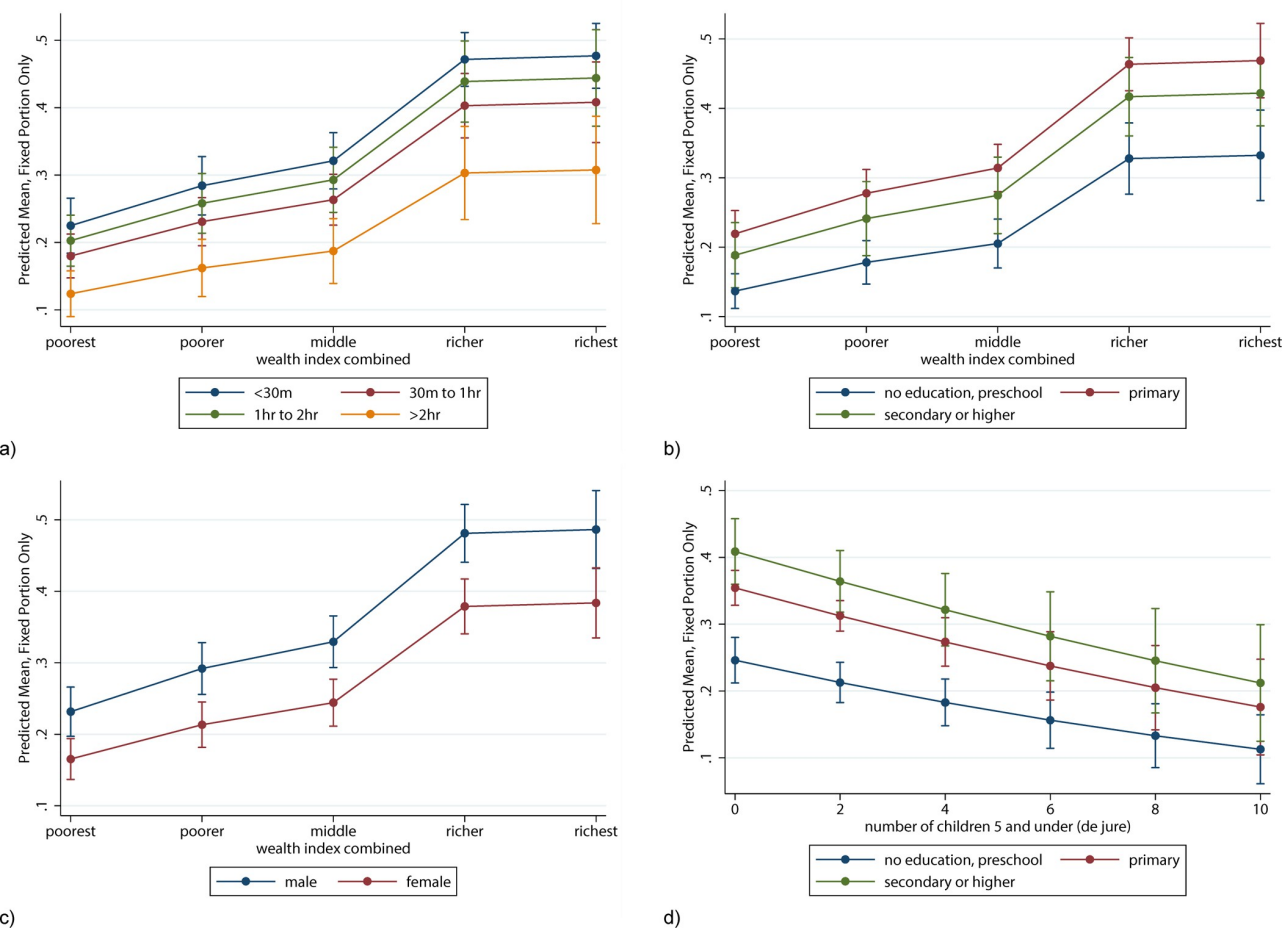
Shows the mean probability to access secondary school for each category of household’s wealth quintile, by (a) travel time to the nearest secondary school; b) level of education of the household’s head; c) child’s sex; d) shows the mean probability to access secondary school for any additional child under the age of five, by level of education of the household’s head.

Adjusted mean predictions for the fixed portion of the model were calculated after running the multilevel logistic model, to compute the probability of accessing secondary school for selected characteristics in the model, holding all the other independent variables in the model at their mean values. The mean probability of a secondary school aged child to attend school was 18% among those residing in a poorest household and at more than 2 hours far away from the nearest secondary school, and 28% among those in poorest households residing within 30 minutes from the nearest school, holding all the other independent variables in the model at their mean values (Fig 3A). The predicted means shown are adjusted to the mean values of all the other independent variables in the full sample. The probabilities grew when looking at children residing in households among the upper two quintiles, reaching almost 50% for those within 30 minutes from the nearest school, and over 30% for those who live as far as 2 hours and above from the nearest secondary school, holding all the other independent variables in the model at their mean values.

Children belonging to a family where the head of household has no education presented consistently lower probabilities to access school across all household wealth quintiles (Fig 3B), with females presenting lower probabilities than males (S3 Fig), compared to those children belonging to a household where the head has at least primary education (Fig 3B). Equally, female children of secondary school age presented consistently lower probabilities of accessing secondary school across all wealth quintiles than their male peers (Fig 3C). Overall, those belonging to wealthier quintiles had higher probabilities to access school, especially those in the wealthiest two quintiles.

For every additional child aged 5 or under, there is a decrease in the probability of accessing school, which is consistent across all the household head education groups (Fig 3D). The attendance probability of a secondary school aged child with no children below the age of 5 present in the household and head of household with no education is equal to 25%, reaching 11% for children with 10 children aged 5 or under. Conversely, for children with household head having at least secondary education, the probability of accessing school ranges between 41% (no children aged 5 and under) and 21% (10 children aged 5 and under), holding all the other independent variables at their mean values.

S4 to S6 Figs, Text and Table show supplementary statistical analyses, including likelihood ratio tests and comparisons between the null and full models, tests for normality of residuals and model calibration.

## Discussion

Ensuring that all children and adolescents have equal opportunities to access and receive adequate education is key to the achievement of Sustainable Development Goal (SDG) 4. The roadmap to achieving this goal in Tanzania requires investigating barriers to access and attendance, and what drives the high secondary out-of-school rates in the country. The results of this study show the importance of looking at individual and household characteristics, such as household wealth, parent’s and head of household’s levels of education and age, age of child, number of children under the age of 5, place of residence and gender as they all affect secondary school attendance in Tanzania. Previous research corroborates these findings [8, 21, 27, 57], suggesting that policies aiming at reducing non-attendance to secondary school should firstly aim at alleviating barriers such as household wealth disadvantages, poverty and gender disparities and at promoting education for the entire population, by introducing sustainable reforms and reducing inequalities. Burke and Beegle (2004) highlighted how the focus of policies should be on demand for schooling within the context of the household, more than on the supply side. Interventions aiming at increasing awareness and informing about cost-benefits and returns from attending school proved to be successful in increasing school attendance in many settings [21, 26, 27], and can be crucial, especially in contexts where secondary school-age children are employed in child labour. Scholarship programs that alleviate cost burdens on the household would be beneficial on a range of aspects, such as by reducing monetary constraints of families with a high dependency ratio, or where children are older than school-age and are therefore thought to be a higher burden on the family [21, 27]. Programs that use conditional cash transfers (CCT) aiming to facilitate enrolment and regular attendance to education had positive impacts on improving attendance in many contexts [27, 33]. However, despite being one of the most widely implemented demand-side programs, questions arise around cash transfer’s strategies and their sustainability and longer term impacts [34] and the possible creation of social stigma [35], among other issues. Vulnerable and marginalized adolescents and youth, and among those, primarily girls, are the most disadvantaged, where unintended and early pregnancies lead to an incredible loss of educational opportunities through the increase in drop-out rates and to the perpetration of gender disparities [58]. Efforts to promote girls’ safe and equal education and to empower adolescents through education are widely recognized as being a priority to transform society and to end the vicious cycle of poverty [59]. Tanzania abolished secondary school fees in 2015, as part of the SDGs and of the 2000–2015 Education For All campaign [60], and in line with many other low and middle-income countries. However, indirect financial burdens on households such as transport, examination papers, school lunch and extra tuition are still present and represent a barrier to access [61]. Moreover, there is a question concerning how the Country’s experience in expanding access can effectively translate into increase in quality education [19, 23].

A particular focus on the role of physical access, measured by travel time and geography in Tanzania was also investigated, and geographic proximity to a secondary school was not found to have a strong association with non-attendance in Tanzania. Based on multilevel multivariate analysis, adolescents living more than 2 hours away from the nearest secondary school (calculated as a combination of walking across land cover and motorised travel along major roads), compared to those living within 30 minutes distance, presented a reduced likelihood of attending secondary school. However, only 9% of the sampled population was located more than 2 hours away from the nearest secondary school, indicating that across Tanzania improvements to increase physical access to schools are still needed. These findings were corroborated by previous studies where school location was not among the most influencing factors [28], although the relationship between location and distance with school access and attendance was found to be strongly significant in other studies in low-and-middle income countries [21, 27]. Geographic proximity to a school alone is insufficient to determine school attendance, and other factors may arise hindering access and attendance, including the quality of services offered, and safety of the environment, especially regarding sexual harassment and abuse [19]. Interventions such as offering funds to purchase bicycles to secondary school year girls were successful in India, and led to a consistent increase in enrolment rates especially among girls living more than three kilometres from the nearest secondary school [62]. Finally, results suggested that residing in certain regions rather than others increased the likelihood of access, therefore part of the variability in access is actually explained by geography.

Pupil to qualified teacher ratio (PQTR) in Tanzania, which was used to give some indication of schools’ characteristics, did not seem to be statistically significant in explaining attendance to school. Results from this work were in line with some previous work [21]. However, maps presented in this work showed interesting patterns of variability of the ratio across the country, together with some geographic clustering and a North-South divide. This suggested that the PQTR indicator, together with other indicators for school characteristics and quality could be valid information for future analysis, where more data were available. Although PQTR has previously been used as a measure of school quality [21], absenteeism constitutes a significant barrier to achieving quality education in both primary and secondary schools, including in Tanzania, where teachers’ attendance remains a challenge [63]. Reducing teachers’ absenteeism through interventions that give incentives to teachers to attend can significantly increase the quality of learning of students [26, 64], contribute to pupils’ learning achievements and eventually lead to broader socio-economic gains [63].

There were some limitations in the study undertaken. This study focused mainly on the out-of-school rates based on the adjusted net attendance rates for all children of official secondary school age, which is one aspect of education. The net attendance rate derived from the household surveys may be subject to reporting and estimation errors, especially where the DHS survey samples are not powered to estimate education-related indicators. Moreover, the construction of the travel time surface is subject to a degree of subjectivity of travel speeds which in reality could vary. Additionally, variations in sizes of schools are not explicitly captured in this analysis, and there location may or may not be influenced by the size of underlying population density impacting estimation of accessibility. Some sources of errors contributed to the uncertainty of the model. The introduction of cluster location random displacement can introduce uncertainty to the modelled relationships when extracting cluster level values, although in general, studies have shown that the impact of displacement is considered to be modest [65, 66]. This potential error was mitigated by extracting average values through a defined buffer around the survey points [67]. Moreover, an additional source of bias can derive by the fact that some DHS clusters points fall outside the catchment of the school, although this work did not aim at investigating these catchments. The analysis of catchment in secondary school education is complex and multidimensional: for example, some pupils may prefer to travel long distances to access boarding schools which offer a wide variety of facilities. Finally, the 2015–16 Tanzania Demographic and Health Survey data, including information about school attendance rates for secondary school age population, and a selection of background information on parents, head of households and households status, were the latest available nationally representative household survey data at the time of the study. While results from this study provide a comprehensive picture of factors associated with school attendance, availability of more recent data may have better captured the current situation of the Country.

To complete the picture of out-of-school, participation, and retention, future work may include focusing on other rates such as gross attendance rates, school drop-out, transition from primary to secondary school, as well as on other age groups. Moreover, information like outcome of school completion as well as data availability on learning outcomes can be important aspects too. Future work can also focus on combining individual, household and access characteristics with data on quality of provisions, costs, absenteeism and other school level indicators such as type of school (public or private), total number of enrolled students, split by age and gender where possible, for all low- and middle-income countries. Future directions may also include investigating the relationships between school access and health, and the role that schools play beyond education, as entities that foster the support and protection of children, adolescents, and young people. Moreover, exploring additional methods to investigate the geographical variation of school access and attendance at lower geographical scales could also be undertaken.

## Supporting information

S1 Fig Distribution of the pupil-qualified teacher ratio (PQTR) in Tanzania. (TIF)

S2 Fig Regions in mainland Tanzania. (TIF)

S3 Fig Mean probability to access secondary school for males and females children, by level of education of the household’s head, and 95% confidence intervals. (TIF)

S4 Fig Caterpillar plots showing ranked estimated level 2 residuals for all clusters in the sample in Tanzania. (TIF)

S5 Fig QQplot: Check for normality of residuals assumption for the main household models. (TIF)

S6 Fig Calibration of the model’s predictions plot for goodness of fit of the model. (TIF)

S1 Table DHS variable descriptions. (DOCX)

S2 Table Summary statistics of DHS variables and contextual variables in Tanzania. (DOCX)

S3 Table Factors associated with school attendance. (DOCX)

S4 Table Multilevel-multivariate analysis of drivers of school attendance. Full results and additional statistics.
(DOCX)

S5 Table Multilevel-multivariate analysis of drivers of school attendance. (DOCX)

S6 Table Multilevel-multivariate analysis of drivers of school attendance, using mother and father’s information and excluding information about household head for Tanzania (2015–16). Full results and additional statistics.
(DOCX)

S7 Table Multilevel-multivariate analysis of drivers of school attendance, using mother and father’s information and excluding information about household head for Tanzania (2015–16). (DOCX)

S8 Table Likelihood ratio tests for null against full models. Tanzania DHS2015-16.
(DOCX)

S1 Text Distribution of the pupil-qualified teacher ratio (PQTR) in Tanzania [46]. (DOCX)

S2 Text QQplot: Check for normality of residuals assumption for the main household models. (DOCX)

S3 Text Calibration of the model’s predictions plot for goodness of fit of the model. (DOCX)
