# Supplementary figures and images for: Understanding factors associated with attending secondary school in Tanzania using household survey data
Source: PLoS One. 2022 Feb 25;17(2):e0263734. doi: 10.1371/journal.pone.0263734 (PMC8880958; doi:10.1371/journal.pone.0263734)

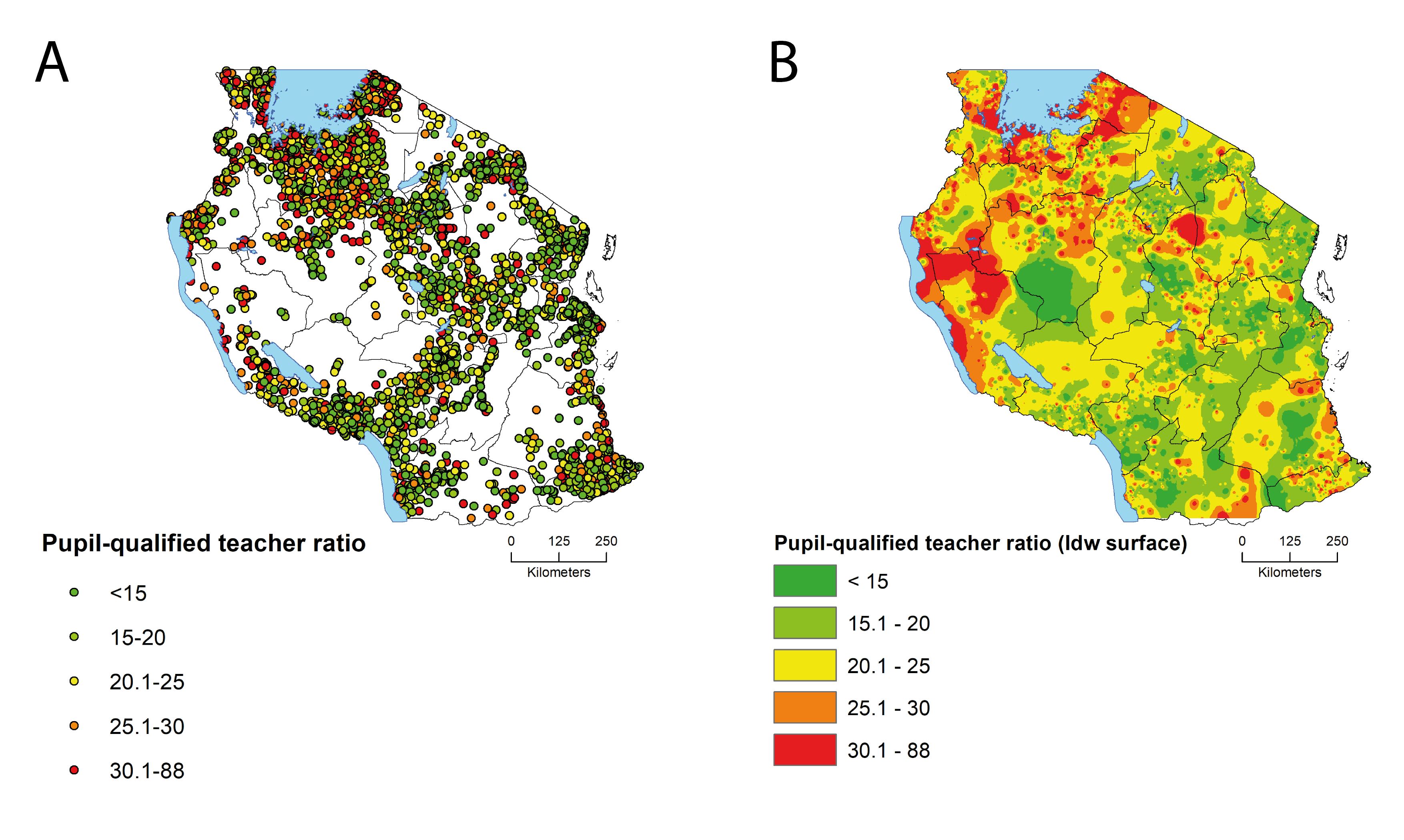

Supplement: S1 Fig — (TIF) [file pone.0263734.s001.tif]

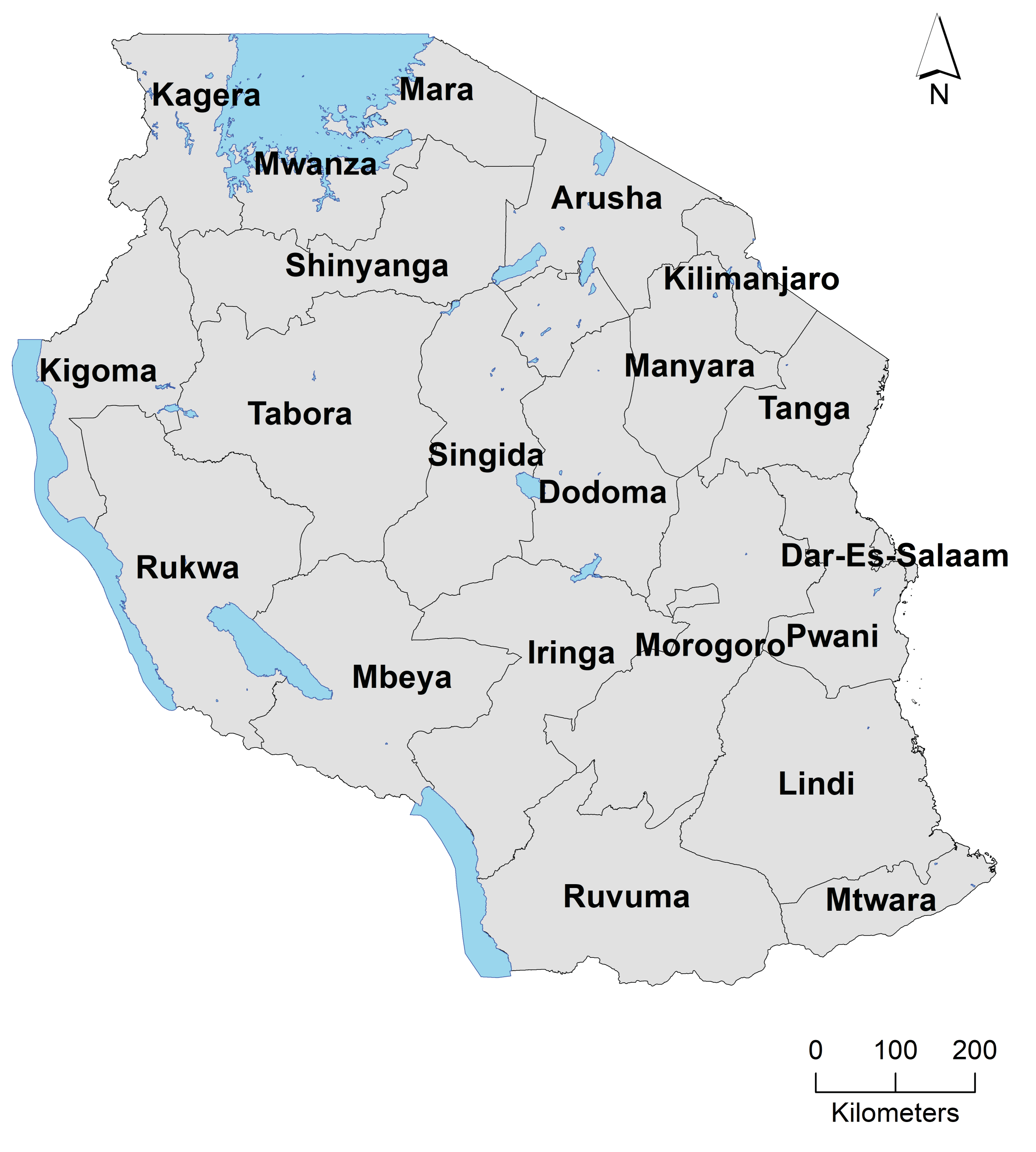

Supplement: S2 Fig — (TIF) [file pone.0263734.s002.tif]

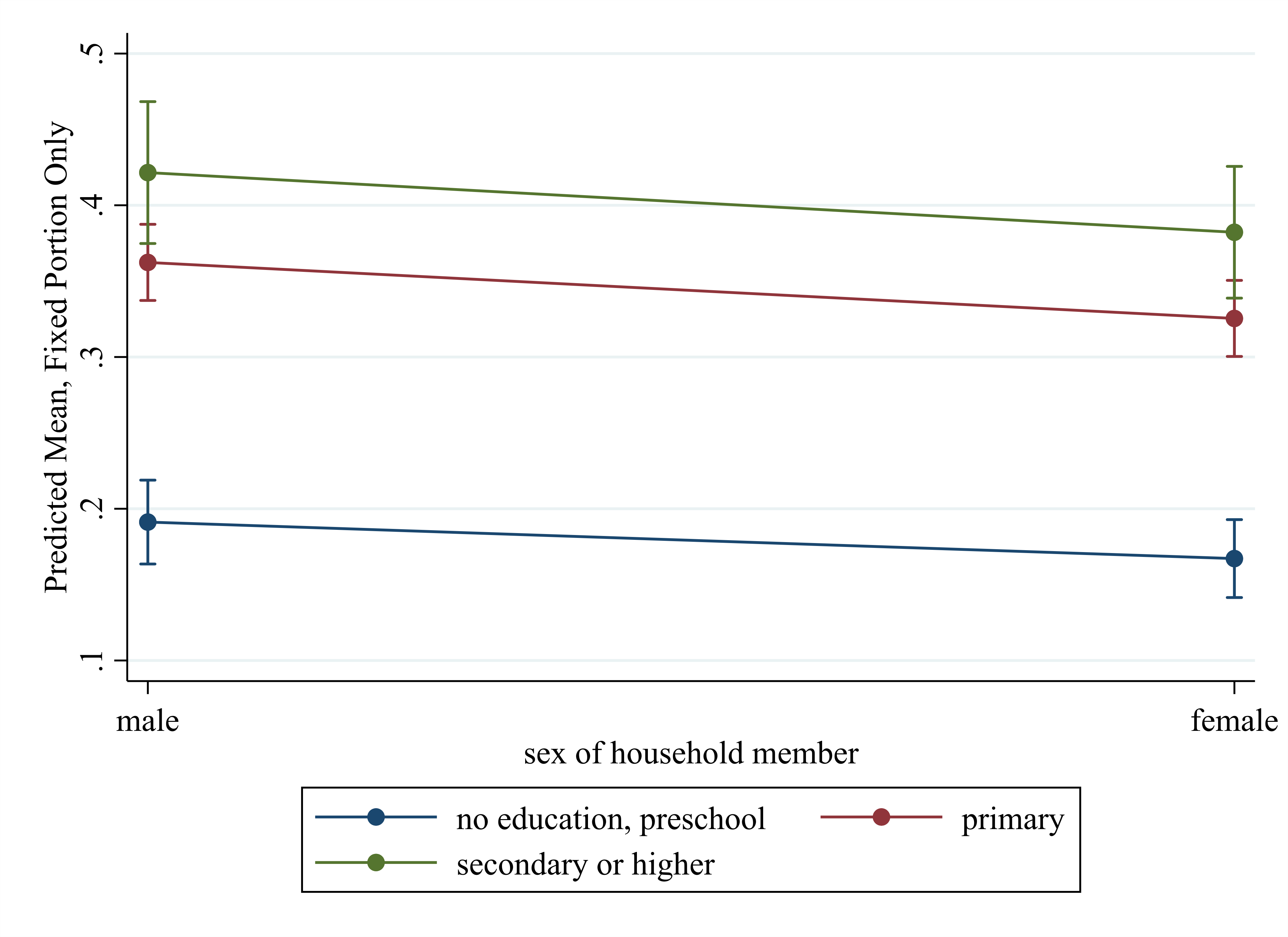

Supplement: S3 Fig — (TIF) [file pone.0263734.s003.tif]

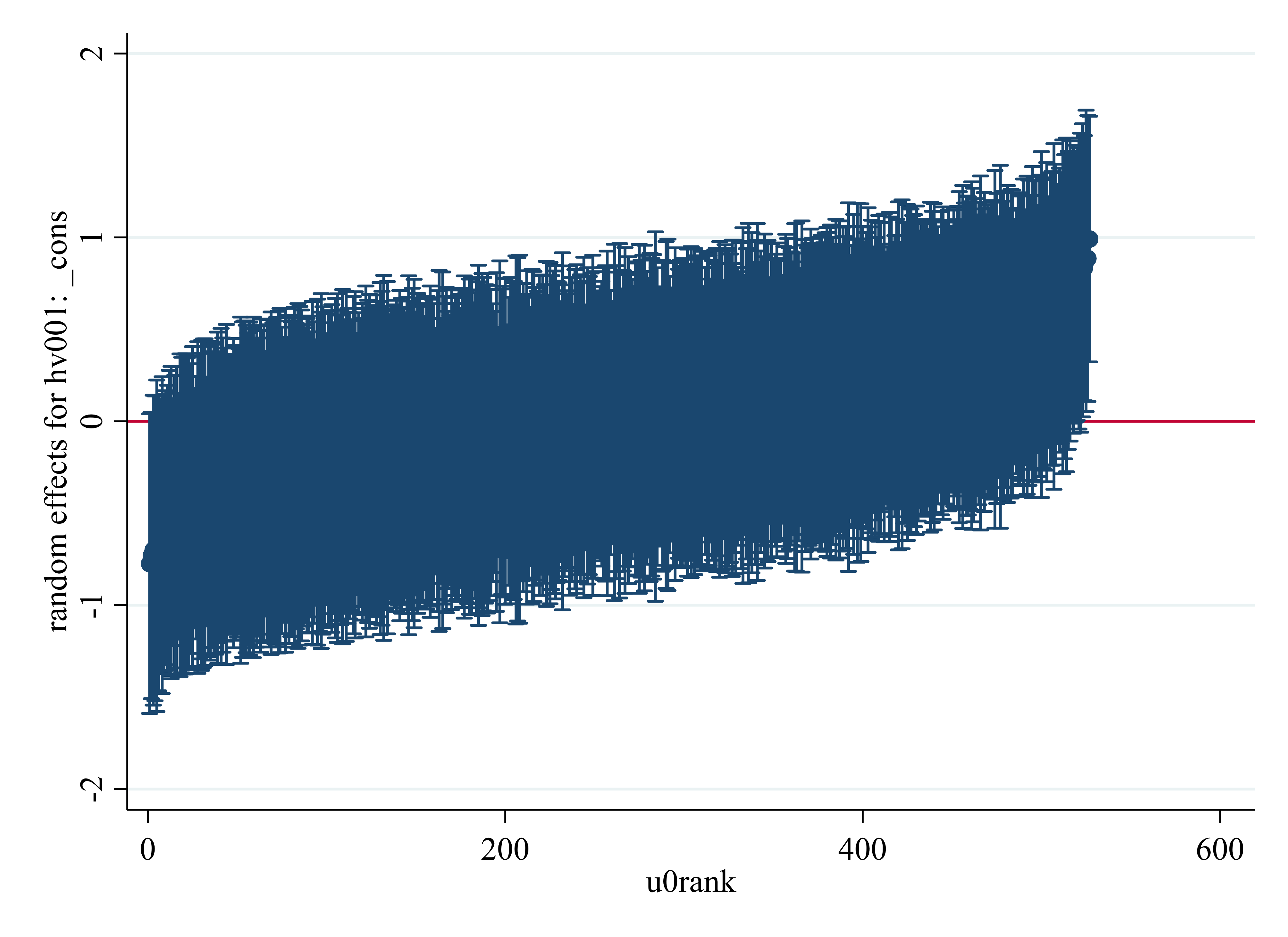

Supplement: S4 Fig — (TIF) [file pone.0263734.s004.tif]

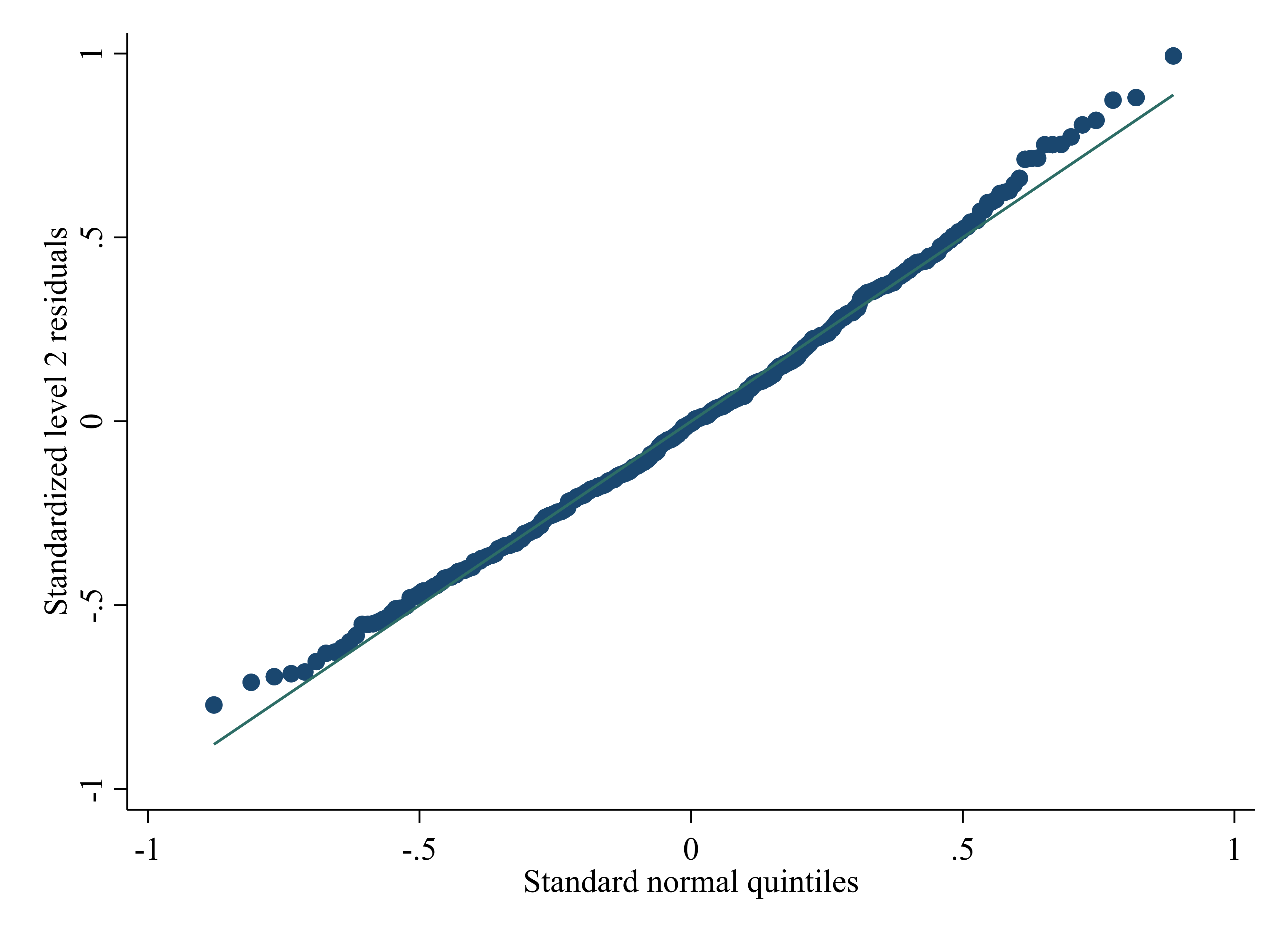

Supplement: S5 Fig — (TIF) [file pone.0263734.s005.tif]

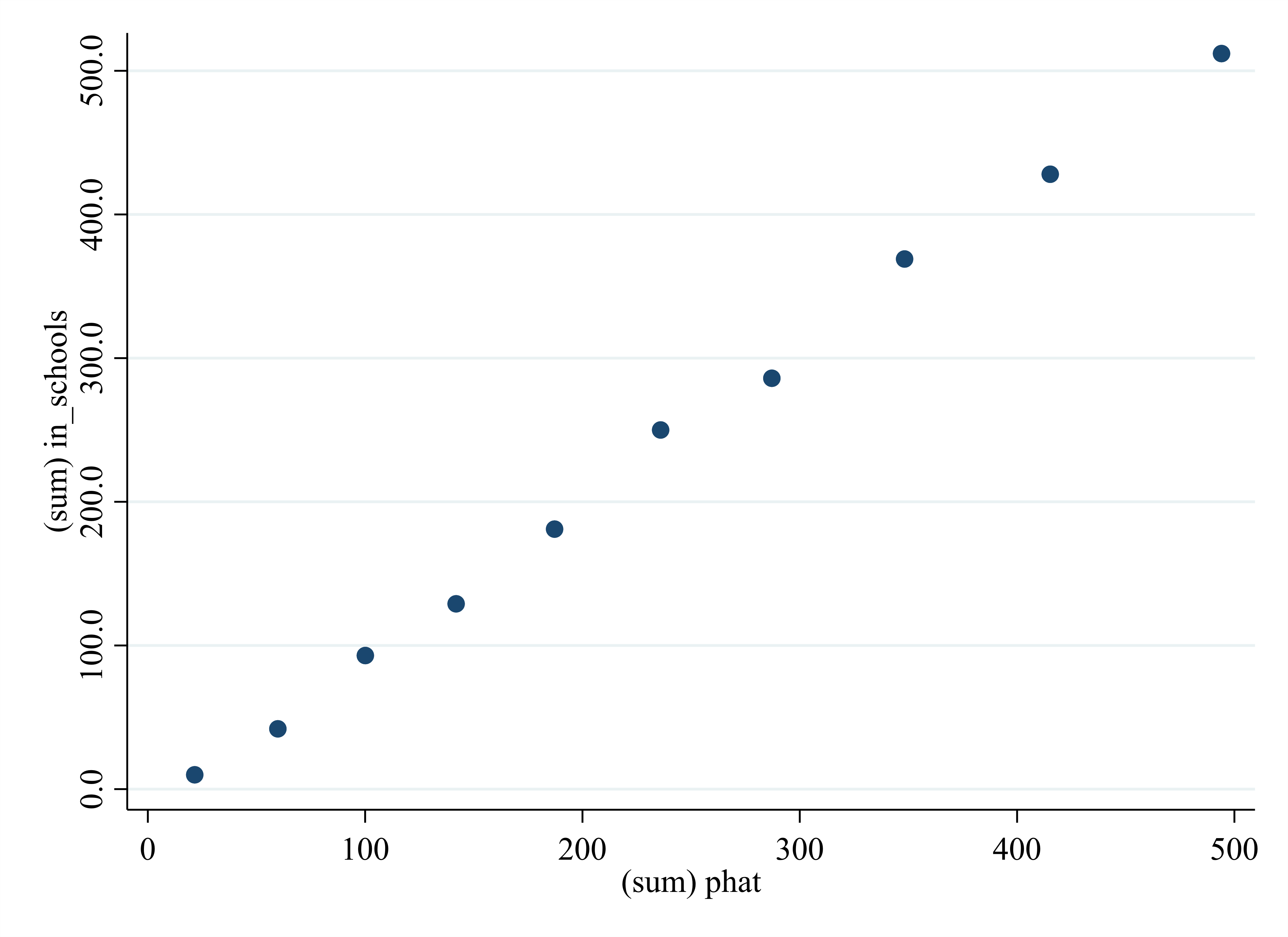

Supplement: S6 Fig — (TIF) [file pone.0263734.s006.tif]
