# Supplementary material for: Understanding factors associated with attending secondary school in Tanzania using household survey data
Source: PLoS One. 2022 Feb 25;17(2):e0263734. doi: 10.1371/journal.pone.0263734 (PMC8880958; doi:10.1371/journal.pone.0263734)
Supplement: S1 Table — (DOCX) [file pone.0263734.s007.docx]

# SI.2 Table: DHS variable descriptions.

A summary of variables assembled from Demographic Health Surveys (DHS) in Tanzania (2015-16). The table includes contextual variables analysed differently.

| **Children (youth 14-19 years), Men (15-54 years), Women (15-49 years) and household level variables** |  |
| --- | --- |
| **Adjusted net attendance rate for secondary school** | Variable hv122 in DHS data tables. Categories: Y=chid attending secondary school, N=secondary school age child not attending. |
| **Province** | The variable reports the de facto region (or province, depending on the country) of residence. Variable hv024. |
| **Place of residence** | Urban or Rural. Variable hv025. |
| **Household Wealth Index** | The wealth index is a composite measure of a household's cumulative living standard based on assets ownership. The wealth index is calculated using data on a household’s ownership of selected assets, such as televisions and bicycles; materials used for housing construction; and types of water access and sanitation facilities. The index is computed using principal components analysis, and the first factor resulting from the analysis places individual households on a continuous scale of relative wealth. DHS groups interviewed households into five wealth quintiles: richest, richer, middle, poorer, poorest. Variable hv190. |
| **Sex of child** | Sex of child as reported by the respondent (male or female). Variable hv104. |
| **Age of child** | Age of child as reported by the respondent. Variable hv105. A squared term for age has been added to the final regression model as age is not linearly related to the outcome. |
| **Adopted/foster child** | As reported by the respondent. Derived from variable hv101 “relationship to head”. Categories: yes=adopted or foster, no=biological child. |
| **Sex of household head** | Self-reported sex of the head of the household: male or female. Variable hv219. |
| **Age of household head** | Self-reported age of the head of the household. Variable hv220. Categorised: <30 years; 30 to 40 years; 41 to 50 years; >50 years. |
| **Highest level of education attained by the head** | Self-reported level of education completed. Categories include: no education, primary, secondary, higher. Variable hv106. |
| **Household ownership of land for agriculture** | Self-reported by the respondent. Categories yes=household owns land; no=household does not own land. Variable hv244. |
| **Household owns livestock, herds or farm animals** | Self-reported by the respondent. Categories yes=household owns livestock; no=household does not own livestock. Variable hv246. |
| **Number of household members** | Self-reported by respondent. Variable hv009. |
| **Number of children under the age of 5** | Self-reported by respondent. Variable hv014. |
| **Number of rooms used for sleeping in the household** | Surveyor observation and respondent reporting. Variable hv216. |
| **Age of mother** | Self-reported by respondent. Information extracted from multiple recode files, and linked to children (variables v012 and hml16). |
| **Mother's highest educational attainment** | Self-reported by respondent. Categories include: No education/preschool, Primary, Secondary, Higher. Information extracted from multiple recode files, and linked to children (variables v106 and hv116). |
| **Mother's mean educational level in single years** | Self-reported by respondent. Information extracted from multiple recode files, and linked to children (variables v133 and hv108). |
| **Marital status** | Current marital status of the respondent. Categories include never married nor in union, married/in union/living together, widowed, divorced, no longer in relationship. Variable v501. |
| **Mean age of father** | Self-reported by respondent. Information extracted from multiple recode files, and linked to children (variables hv105 and mv012). |
| **Father's highest educational attainment** | Reported by respondent. Categories include: No education, Primary, Secondary, Higher. Information extracted from multiple recode files, and linked to children (variables hv105 and mv012). |
| **Father's educational level in single years** | Self-reported by respondent. Information extracted from multiple recode files, and linked to children (variables hv108 and mv133). |
| **Husband/partner's occupation** | Reported by respondent. Categories include: did not work, professional/technical/managerial, clerical, agricultural - self employed, agricultural – employee, household and domestic, services, skilled manual, unskilled manual. Variable v705. |
| **Contextual variables (cluster level)** |  |
| **Travel time to school** | Mean travel time to secondary school by cluster (PSU) |
| **Pupil to qualified teacher ratio (available for TZ only)** | Mean pupil to qualified teacher ration by cluster (PSU) |

For more information about the DHS indicator variables, consult [43] and www.dhsprogram.com/Data/Guide-to-DHS-Statistics
