# Supplementary material for: Understanding factors associated with attending secondary school in Tanzania using household survey data
Source: PLoS One. 2022 Feb 25;17(2):e0263734. doi: 10.1371/journal.pone.0263734 (PMC8880958; doi:10.1371/journal.pone.0263734)
Supplement: S2 Table — (DOCX) [file pone.0263734.s008.docx]

# SI.3 Table: Summary statistics of DHS variables and contextual variables in

# Tanzania.

Socio-demographic and background characteristics of children of secondary school age in Tanzania 2015-16 DHS

| ***Children 14-19 years, Men (15-54 years), Women (15-49 years) and household level variables*** |  |  |
| --- | --- | --- |
|  | **% (weighted) / mean** | **N (non-weighed)** |
| ***Region*** |  |  |
| *Dodoma* | 4.5 | 199 |
| *Arusha* | 2.9 | 176 |
| *Kilimanjaro* | 2.5 | 185 |
| *Tanga* | 5.4 | 255 |
| *Morogoro* | 4.2 | 183 |
| *Pwani* | 2.1 | 177 |
| *Dar es salaam* | 9.4 | 372 |
| *Lindi* | 1.8 | 182 |
| *Mtwara* | 2.5 | 155 |
| *Ruvuma* | 2.7 | 201 |
| *Iringa* | 1.9 | 191 |
| *Mbeya* | 6.0 | 195 |
| *Singida* | 2.9 | 249 |
| *Tabora* | 6.7 | 377 |
| *Rukwa* | 2.0 | 212 |
| *Kigoma* | 4.4 | 291 |
| *Shinyanga* | 4.1 | 310 |
| *Kagera* | 5.1 | 251 |
| *Mwanza* | 7.7 | 325 |
| *Mara* | 3.7 | 277 |
| *Manyara* | 2.7 | 224 |
| *Njombe* | 1.2 | 155 |
| *Katavi* | 0.9 | 245 |
| *Simiyu* | 4.9 | 413 |
| *Geita* | 5.0 | 397 |
| *Kaskazini unguja* | 0.5 | 212 |
| *Kusini unguja* | 0.3 | 198 |
| *Mjini magharibi* | 1.4 | 352 |
| *Kaskazini pemba* | 0.5 | 206 |
| *Kusini pemba* | 0.4 | 226 |
| ***Type of place of residence*** |  |  |
| *Urban* | 33.0 | 2130 |
| *Rural* | 67.0 | 5261 |
| ***Household wealth Index*** |  |  |
| *poorest* | 17.8 | 1235 |
| *poorer* | 17.2 | 1243 |
| *middle* | 18.8 | 1419 |
| *richer* | 21.3 | 1705 |
| *richest* | 24.9 | 1789 |
| ***Sex of household head*** |  |  |
| *Male* | 76.6 | 5717 |
| *Female* | 23.4 | 1674 |
| ***Mean age of household head (SD)*** | 48.7 (14.088) |  |
| ***Head highest level of education attained*** |  |  |
| *no education* | 19.5 | 1559 |
| *primary* | 63.9 | 4457 |
| *secondary* | 13.3 | 1160 |
| *higher* | 3.2 | 207 |
| *don't know* | 0.0 | 7 |
| ***Sex of child*** |  |  |
| *male* | 49.2 | 3663 |
| *female* | 50.8 | 3728 |
| ***Mean age of child (SD)*** | 16.3 (1.728) |  |
| ***Adopted/foster child*** |  |  |
| *Yes* | 2.7 | 218 |
| *No* | 97.3 | 7173 |
| ***Household owns land for agriculture*** |  |  |
| *Yes* | 68.6 | 4972 |
| *No* | 31.4 | 2419 |
| ***Household owns livestock, herds or farm animals*** |  |  |
| *Yes* | 67.9 | 5047 |
| *No* | 32.1 | 2344 |
| ***Mean number of household members (SD)*** | 7.5 (3.97) |  |
| ***Mean number of children under 5 (SD)*** | 1.15 (1.35) |  |
| ***Mean number of rooms for sleeping (SD)*** | 3 (1.34) |  |
| ***Mean age of mother (SD)†*** | 42.9(6.61) |  |
| ***Mother's highest educational attainment†*** |  |  |
| *No education, preschool* | 24.4 | 954 |
| *Primary* | 69.4 | 2280 |
| *Secondary* | 5.8 | 351 |
| *Higher* | 0.5 | 4 |
| ***Marital Status†*** |  |  |
| *Never married nor in union* | 1.1 | 33 |
| *Married/in union* | 80.8 | 2960 |
| *Widow* | 9.2 | 300 |
| *Divorced* | 5.2 | 188 |
| *No longer in relationship* | 3.7 | 108 |
| ***Mother's mean educational level in single years (SD)†*** | 5.15(3.43) |  |
| ***Mean age of father (SD)††*** | 49.7(8.93) |  |
| ***Father's highest educational attainment††*** |  |  |
| *No education, preschool* | 15.0 | 399 |
| *Primary* | 73.9 | 1577 |
| *Secondary* | 9.5 | 284 |
| *Higher* | 1.7 | 26 |
| *Don't know* | 0.0 | 2 |
| ***Father's mean educational level in single years (SD)††*** | 6.14(4.6) |  |
| ***Husband/partner's occupation†††*** |  |  |
| *did not work* | 1.1 | 30 |
| *professional/technical/managerial* | 5.3 | 120 |
| *clerical* | 0.5 | 16 |
| *agricultural - self employed* | 57.5 | 1384 |
| *agricultural - employee* | 5.0 | 165 |
| *household and domestic* | 1.1 | 33 |
| *services* | 5.7 | 134 |
| *skilled manual* | 12.3 | 301 |
| *unskilled manual* | 11.3 | 253 |
| *don't know* | 0.2 | 4 |
|  |  |  |
| ***Total*** | 100 | 7391 |
|  |  |  |
| ***Contextual variables (cluster level / PSU)*** |  |  |
|  |  | **N** |
|  |  |  |
| *Mean travel time to secondary school by PSU (SD)* | 51.9(66.5) | 527 PSUs^ |
| *Mean Pupil to qualified teacher ratio (PQTR) by PSU (SD)* | 23.1(6.36) | 527 PSUs^ |
| *Mean Pupil to teacher ratio (PTR) by PSU (SD)* | 22.8(6.26) | 527 PSUs^ |

†Total number of valid observations available for mothers' sample for Tanzania N=3,589††Total number of valid observations available for father' sample N=2,288

†††Responses from women interviews. Total number of valid observations N=2,440

^No data for Zanzibar and islands

*1,400 missing values

The following regions are located on the island of Zanzibar: Kaskazini unguja, Kusini unguja, Mjini magharibi. The following regions are located on the island of Pemba: Kaskazini pemba, Kusini pemba. These were included in descriptive and bivariate analyses only.

Based on the 2015-16 Tanzania DHS survey, almost 65% of children of secondary school age were not attending school. Table SI.3 shows summary statistics for selected socio-demographic and background characteristics of children of secondary school age in Tanzania 2015-16 DHS, including information on children (14-19 years), men (15-54 years), women (15-49 years) and household level variables, presenting great variation across background characteristics. Summary statistics of contextual variables were also reported.

Bivariate analysis for factors associated with secondary school attendance
