# Supplementary material for: Understanding factors associated with attending secondary school in Tanzania using household survey data
Source: PLoS One. 2022 Feb 25;17(2):e0263734. doi: 10.1371/journal.pone.0263734 (PMC8880958; doi:10.1371/journal.pone.0263734)
Supplement: S3 Table — (DOCX) [file pone.0263734.s009.docx]

# SI.4 Table: Factors associated with school attendance.

The distribution of individual, household-level and background characteristics, by school attendance status for children of adolescence group or at youth level in Tanzania (DHS 2015-16).

|  | | ***% Not attending [95%CI] (or mean and SD^)*** | ***% Attending [95%CI] (or mean and SD^)*** | ***P-value (95% CI)1*** |
| --- | --- | --- | --- | --- |
| ***Tanzania (Mainland)*** | **Region** | | | <0.001* |
|  | Dodoma | 70.4 [60.8,78.4] | 29.7 [21.6,39.2] |  |
|  | Arusha | 58.8 [46.99,69.71] | 41.2 [30.29,53.01] |  |
|  | Kilimanjaro | 42.8 [33.75,52.35] | 57.2 [47.65,66.25] |  |
|  | Tanga | 61.2 [54.68,67.34] | 38.8 [32.66,45.32] |  |
|  | Morogoro | 64.8 [53.3,74.72] | 35.3 [25.28,46.7] |  |
|  | Pwani | 58.5 [50.15,66.3] | 41.6 [33.7,49.85] |  |
|  | Dar es salaam | 60.2 [55.24,64.98] | 39.8 [35.02,44.76] |  |
|  | Lindi | 72.1 [62.66,79.89] | 27.9 [20.11,37.34] |  |
|  | Mtwara | 74.7 [65.69,81.97] | 25.3 [18.03,34.31] |  |
|  | Ruvuma | 63.0 [54.89,70.35] | 37.1 [29.65,45.11] |  |
|  | Iringa | 44.2 [34.99,53.82] | 55.8 [46.18,65.01] |  |
|  | Mbeya | 64.3 [49.42,76.82] | 35.7 [23.18,50.58] |  |
|  | Singida | 53.3 [43.53,62.86] | 46.7 [37.14,56.47] |  |
|  | Tabora | 74.2 [66.43,80.66] | 25.8 [19.34,33.57] |  |
|  | Rukwa | 76.8 [68.43,83.41] | 23.3 [16.59,31.57] |  |
|  | Kigoma | 65.7 [53.79,75.98] | 34.3 [24.02,46.21] |  |
|  | Shinyanga | 77.5 [71.64,82.45] | 22.5 [17.55,28.36] |  |
|  | Kagera | 51.3 [43.9,58.6] | 48.7 [41.4,56.1] |  |
|  | Mwanza | 60.0 [54.3,65.51] | 40.0 [34.49,45.7] |  |
|  | Mara | 60.1 [48.71,70.54] | 39.9 [29.46,51.29] |  |
|  | Manyara | 65.8 [54.21,75.8] | 34.2 [24.2,45.79] |  |
|  | Njombe | 69.5 [55.88,80.41] | 30.5 [19.59,44.12] |  |
|  | Katavi | 70.2 [57.15,80.63] | 29.8 [19.37,42.85] |  |
|  | Simiyu | 61.5 [53.93,68.6] | 38.5 [31.4,46.07] |  |
|  | Geita | 58.4[51.76,64.69] | 41.6 [35.31,48.24] |  |
|  | Kaskazini unguja | 39.0 [34.68,43.51] | 61.0 [56.49,65.32] |  |
|  | Kusini unguja | 42.8 [35.36,50.54] | 57.2 [49.46,64.64] |  |
|  | Mjini magharibi | 34.1 [28.34,40.35] | 65.9 [59.65,71.66] |  |
|  | Kaskazini pemba | 51.2 [41.74,60.48] | 48.9 [39.52,58.26] |  |
|  | Kusini pemba | 41.6 [33.68,49.88] | 58.5 [50.12,66.32] |  |
|  | **Type of place of residence** | | | |
|  | Urban | 54.4 [51.6, 57.2] | 45.6 [42.8, 48.4] | <0.001* |
|  | Rural | 66.1 [63.7, 68.3] | 34.0 [31.6, 36.3] |  |
|  | **Household wealth Index** | | | |
|  | poorest | 78.6 [75.03,81.78] | 21.4 [18.22,24.97] | <0.001* |
|  | poorer | 70.2 [66.54,73.65] | 29.8 [26.35,33.46] |  |
|  | middle | 64.2 [60.67,67.53] | 35.8 [32.47,39.33] |  |
|  | richer | 52.8 [49.29,56.35] | 47.2 [43.65,50.71] |  |
|  | richest | 51.5 [48.75,54.33] | 48.5 [45.67,51.25] |  |
|  | **Sex of household head** | | | |
|  | Male | 62.0 [59.98,64.05] | 38.0 [35.95,40.02] | 0.6333 |
|  | Female | 62.8 [59.75,65.83] | 37.2 [34.17,40.25] |  |
|  | **Mean age of household head (SD)** | 47.3 (14.7) | 50.6 (12.4) | <0.001^* |
|  | **Head highest level of education attained** |  |  |  |
|  | no education | 74.2 [70.99,77.21] | 25.8 [22.79,29.01] | <0.001* |
|  | primary | 60.7 [58.56,62.77] | 39.3 [37.23,41.44] |  |
|  | secondary | 52.6 [50.84,57.11] | 47.4 [42.89,49.16] |  |
|  | higher | 59.9 | 40.1 |  |
|  | don't know | 72.9 | 27.1 |  |
|  | **Sex of child** | | | |
|  | male | 59.3 [56.95,61.59] | 40.7 [38.41,43.05] | <0.001* |
|  | female | 65.1 [62.78,67.28] | 34.9 [32.72,37.22] |  |
|  | **Mean age of child (SD)** | 16.8 (1.6) | 15.6 (1.6) | <0.001^* |
|  | **Adopted/foster child** | | | |
|  | Yes | 65.3 [56.4,73.17] | 34.8 [26.83,43.6] | 0.4857 |
|  | No | 62.1 [60.25,63.99] | 37.9 [36.01,39.75] |  |
|  | **Household owns land for agriculture** | | | |
|  | Yes | 63.5 [61.12,65.8] | 36.5 [34.2,38.88] | 0.0211* |
|  | No | 59.5 [56.83,62.02] | 40.6 [37.98,43.17] |  |
|  | **Household owns livestock, herds or farm animals** | | | |
|  | Yes | 62.2 [60.06,64.34] | 37.8 [35.66,39.94] | 0.9979 |
|  | No | 62.2 [59.56,64.81] | 37.8 [35.19,40.44] |  |
|  | **Mean number of household members (SD)** | 7.53 (4.2) | 7.47 (3.5) | 0.7009 |
|  | **Mean number of children under 5 (SD)** | 1.25 (1.4) | 0.98 (1.3) | <0.001* |
|  | **Mean number of rooms for sleeping (SD)** | 2.9 (1.4) | 3.1 (1.3) | <0.001* |
|  | **Mean age of mother (SD)†** | 43.3 (6.7) | 42.6 (6.5) | 0.001^* |
|  | **Mother's highest educational attainment†** | | | |
|  | No education, preschool | 65.5 [61.19,69.66] | 34.5 [30.34,38.81] | <0.001* |
|  | Primary | 48.5 [45.79,51.22] | 51.5 [48.78,54.21] |  |
|  | Secondary | 15.6 [10.73,22.14] | 84.4 [77.86,89.27] |  |
|  | Higher | 36.8 [17.3,61.87] | 63.2 [38.13,82.7] |  |
|  | **Marital Status†** | | | |
|  | Never married nor in union | 43.5 [25.48,63.46] | 56.5 [36.54,74.52] | <0.001* |
|  | Married/in union | 49.0 [46.37,51.71] | 51.0 [48.29,53.63] |  |
|  | Widow | 59.5 [51.75,66.84] | 40.5 [33.16,48.25] |  |
|  | Divorced | 62.2 [53.99,69.71] | 37.8 [30.29,46.01] |  |
|  | No longer in relationship | 51.0 [39.04,62.75] | 49.1 [37.25,60.96] |  |
|  | **Mother's mean educational level in single years (SD)†** | 4.4 (3.3) | 6 (3.5) | <0.001^* |
|  | **Mean age of father (SD)††** | 50.3 (9.4) | 49.2 (8.5) | 0.0152^ |
|  | **Father's highest educational attainment††** | | | |
|  | No education, preschool | 65.1 [59.25,70.61] | 34.9 [29.39,40.75] | <0.001* |
|  | Primary | 41.5 [38.46,44.61] | 58.5 [55.39,61.54] |  |
|  | Secondary | 15.1 [9.788,22.53] | 84.9 [77.47,90.21] |  |
|  | Higher | 19.1 [5.698,48.09] | 80.9 [51.91,94.3] |  |
|  | Don't know | 0.0 | 100.0 |  |
|  | **Father's mean educational level in single years (SD)††** | 5 (3.3) | 7 (5.3) | <0.001^* |
|  | **Husband/partner's occupation†††** | | | |
|  | did not work | 66.3 [45.06,82.48] | 33.7 [17.52,54.94] | <0.001* |
|  | professional/technical/managerial | 22.0 [12.95,34.86] | 78.0 [65.14,87.05] |  |
|  | clerical | 15.2 [2.078,60.16] | 84.8 [39.84,97.92] |  |
|  | agricultural - self employed | 56.4 [52.8,59.92] | 43.6 [40.08,47.2] |  |
|  | agricultural - employee | 41.2 [29.43,54.06] | 58.8 [45.94,70.57] |  |
|  | household and domestic | 27.5 [11.52,52.37] | 72.6 [47.63,88.48] |  |
|  | services | 47.1 [36.09,58.41] | 52.9 [41.59,63.91] |  |
|  | skilled manual | 41.4 [34.71,48.5] | 58.6 [51.5,65.29] |  |
|  | unskilled manual | 41.4 [34.42,48.83] | 58.6 [51.17,65.58] |  |
|  | don't know | 49.9 [21.77,78.03] | 50.2 [21.97,78.23] |  |
|  | **Total** | **62.2 [60.37,64.04]** | **37.8 [35.96,39.63]** |  |
|  | **Contextual variables (cluster level / PSU)** | | | |
|  | Mean travel time to secondary school by PSU (SD) | 57 (73.0) | 43.1 (52.8) | <0.001* |
|  | Mean Pupil to qualified teacher ratio (PQTR) by PSU (SD) | 23.2 (6.3) | 23.1 (6.5) | 0.863 |
|  | Mean Pupil to teacher ratio (PTR) by PSU (SD) | 22.8 (6.2) | 22.7 (6.5) | 0.8622 |

^1^ p-values are produced using F test, with a significance level of 0.05. “Svy” command in STATA was used to account for weighting and sampling design.

^^^ calculated using t-test for equality of means, with a significance level of 0.05. “Svy” command in STATA was used to account for weighting and sampling design.

^†^Total number of valid observations available for mothers' sample N=3,589 (Tanzania).

^††^Total number of valid observations available for father' sample N=2,288 (Tanzania).

^†††^Responses from women interviews. Total number of valid observations N=2,440 (Tanzania).

^*^ Statistically significant at 95% confidence interval (p value < 0.05).

The following regions are located on the island of Zanzibar: Kaskazini unguja, Kusini unguja, Mjini magharibi. The following regions are located on the island of Pemba: Kaskazini pemba, Kusini pemba. These were included in descriptive and bivariate analyses only.

Table SI.4 shows results for a bivariate analysis of various indicators by school attendance status in Tanzania. Region, household wealth index, number of children under the age of 5 present in the household, level of education of the household head and level of education of the mother and father were important explanatory variables associated with school attendance in Tanzania. Mean travel time to school, place of residence, marital status and father’s education, partner occupation and age and sex of child were also strongly associated with school attendance.
