# Supplementary material for: Understanding factors associated with attending secondary school in Tanzania using household survey data
Source: PLoS One. 2022 Feb 25;17(2):e0263734. doi: 10.1371/journal.pone.0263734 (PMC8880958; doi:10.1371/journal.pone.0263734)
Supplement: S4 Table — Full results and additional statistics. (DOCX) [file pone.0263734.s010.docx]

# SI.5.1 Table: Multilevel-multivariate analysis of drivers of school

# attendance. Full results and additional statistics.

Odds ratios, standard errors, z-test statistic, p-values and 95% confidence intervals from the two-level logistic regression model of school attendance among children of adolescents or youth-level using DHS data in Tanzania (2015-16 DHS, N=6,197).

|  | **Odds Ratio** | **Std. Err.** | **z** | **P>z** | **95% CI -** | **95% CI +** |
| --- | --- | --- | --- | --- | --- | --- |
| **Place of residence (REF: Urban)** |  |  |  |  |  |  |
| Rural | 0.8339038 | 0.1079409 | -1.4 | 0.161 | 0.6470473 | 1.074721 |
|  |  |  |  |  |  |  |
| **Household wealth index (Poorest)** |  |  |  |  |  |  |
| Poorer | 1.368356 | 0.1545645 | 2.78 | 0.005 | 1.096606 | 1.707449 |
| Middle | 1.630271 | 0.1878482 | 4.24 | < 0.001 | 1.300708 | 2.043335 |
| Richer | 3.076087 | 0.3855901 | 8.96 | < 0.001 | 2.406023 | 3.93276 |
| Richest | 3.14204 | 0.48897 | 7.36 | < 0.001 | 2.31604 | 4.262626 |
|  |  |  |  |  |  |  |
| **Sex of child (REF:male)** |  |  |  |  |  |  |
| female | 0.8489445 | 0.0538603 | -2.58 | 0.01 | 0.7496798 | 0.9613529 |
|  |  |  |  |  |  |  |
| **DHS Region (Dodoma)** |  |  |  |  |  |  |
| Arusha | 1.035974 | 0.3242931 | 0.11 | 0.91 | 0.5609131 | 1.913385 |
| Kilimanjaro | 1.96087 | 0.6053864 | 2.18 | 0.029 | 1.070666 | 3.59123 |
| Tanga | 1.375795 | 0.4064533 | 1.08 | 0.28 | 0.7710493 | 2.454854 |
| Morogoro | 0.8436189 | 0.2644642 | -0.54 | 0.588 | 0.4563574 | 1.559508 |
| Pwani | 1.238708 | 0.3861923 | 0.69 | 0.492 | 0.6723409 | 2.282171 |
| Dar es salaam | 0.7550408 | 0.2247543 | -0.94 | 0.345 | 0.4213003 | 1.35316 |
| Lindi | 0.8007814 | 0.2532519 | -0.7 | 0.482 | 0.4308404 | 1.488372 |
| Mtwara | 0.5467859 | 0.1812141 | -1.82 | 0.069 | 0.2855713 | 1.046936 |
| Ruvuma | 1.081741 | 0.3292087 | 0.26 | 0.796 | 0.5957656 | 1.964135 |
| Iringa | 2.69791 | 0.8224892 | 3.26 | 0.001 | 1.484324 | 4.903725 |
| Mbeya | 1.146157 | 0.3505628 | 0.45 | 0.656 | 0.6293558 | 2.087334 |
| Singida | 2.235265 | 0.6568036 | 2.74 | 0.006 | 1.256651 | 3.975974 |
| Tabora | 1.11951 | 0.3203805 | 0.39 | 0.693 | 0.6388987 | 1.961662 |
| Rukwa | 0.6818665 | 0.2136504 | -1.22 | 0.222 | 0.3689699 | 1.260108 |
| Kigoma | 1.129166 | 0.3293447 | 0.42 | 0.677 | 0.6375099 | 1.999992 |
| Shinyanga | 0.6416406 | 0.1935368 | -1.47 | 0.141 | 0.3552594 | 1.158879 |
| Kagera | 1.948526 | 0.5773021 | 2.25 | 0.024 | 1.090223 | 3.482547 |
| Mwanza | 1.336044 | 0.3848969 | 1.01 | 0.315 | 0.7596278 | 2.349854 |
| Mara | 1.059123 | 0.3149232 | 0.19 | 0.847 | 0.5913534 | 1.896905 |
| Manyara | 1.537636 | 0.4624081 | 1.43 | 0.153 | 0.8528535 | 2.77225 |
| Njombe | 0.5978828 | 0.1985691 | -1.55 | 0.121 | 0.3118276 | 1.146351 |
| Katavi | 1.155859 | 0.3459696 | 0.48 | 0.628 | 0.6428721 | 2.078188 |
| Simiyu | 1.548734 | 0.4310527 | 1.57 | 0.116 | 0.8975643 | 2.672319 |
| Geita | 1.637658 | 0.4623989 | 1.75 | 0.081 | 0.9416368 | 2.848152 |
|  |  |  |  |  |  |  |
| **Age of head of household (REF: >50 years old)** |  |  |  |  |  |  |
| <30 years | 0.1461054 | 0.0254549 | -11.04 | < 0.001 | 0.1038406 | 0.2055725 |
| 30 to 40 | 0.6716272 | 0.0656607 | -4.07 | < 0.001 | 0.5545131 | 0.8134762 |
| 40 to 50 | 1.079303 | 0.0794962 | 1.04 | 0.3 | 0.934218 | 1.246921 |
|  |  |  |  |  |  |  |
| **Highest education attainments of the head (REF: no education)** |  |  |  |  |  |  |
| primary | 1.681105 | 0.1562879 | 5.59 | < 0.001 | 1.401073 | 2.017106 |
| secondary+ | 2.118294 | 0.2854557 | 5.57 | < 0.001 | 1.6266 | 2.75862 |
|  |  |  |  |  |  |  |
| **Travel time to nearest secondary school (<30 min)** |  |  |  |  |  |  |
| Between 30min and 1hr | 0.8877556 | 0.1027155 | -1.03 | 0.303 | 0.7076318 | 1.113729 |
| Between 1hr and 2 hr | 1.043533 | 0.141588 | 0.31 | 0.753 | 0.7998603 | 1.36144 |
| More than 2hr | 0.6797729 | 0.1209387 | -2.17 | 0.03 | 0.4796519 | 0.9633885 |
|  |  |  |  |  |  |  |
| **Number of children under 5** | 0.9099296 | 0.0254037 | -3.38 | 0.001 | 0.861477 | 0.9611072 |
| **Age of child** | 0.3783248 | 0.1622811 | -2.27 | 0.023 | 0.1632093 | 0.8769699 |
| **Age squared** | 1.014804 | 0.0133364 | 1.12 | 0.263 | 0.9889992 | 1.041283 |
| **Pupil to qualified teacher ratio (PQTR)** | 1.014014 | 0.0077046 | 1.83 | 0.067 | 0.9990248 | 1.029228 |
| _cons | 26000.97 | 90638.28 | 2.92 | 0.004 | 28.03733 | 2.41E+07 |
|  |  |  |  |  |  |  |
| **Random-effects Parameters** | **Estimate** | **Std. Err.** |  |  | **95% CI -** | **95% CI +** |
|  |  |  |  |  |  |  |
| **DHS clusters** | 0.2773776 | 0.0542914 |  |  | 0.1890013 | 0.4070782 |
| Note: _cons estimates baseline odds (conditional on zero random effects). | | |  |  |  |  |
