# Supplementary material for: Understanding factors associated with attending secondary school in Tanzania using household survey data
Source: PLoS One. 2022 Feb 25;17(2):e0263734. doi: 10.1371/journal.pone.0263734 (PMC8880958; doi:10.1371/journal.pone.0263734)
Supplement: S5 Table — (DOCX) [file pone.0263734.s011.docx]

# SI.5 Table: Multilevel-multivariate analysis of drivers of school attendance.

Odds ratios and 95% confidence intervals from the two-level logistic regression model of school attendance among children of adolescents or youth-level using DHS data in Tanzania (2015-16 DHS, N=6,197).

| Fixed Effects | *Odds Ratios (95% CI)* |
| --- | --- |
| Place of residence (REF: Urban) |  |
| Rural | 0.83 (0.64,1.07) |
| Household wealth index (Poorest) |  |
| Poorer | 1.37 (1.09,1.71) |
| Middle | 1.63 (1.3,2.04) |
| Richer | 3.01 (2.41,3.93) |
| Richest | 3.14 (2.32,4.26) |
| DHS Region (Dodoma) |  |
| Arusha | 1.04 (0.56,1.91) |
| Kilimanjaro | 1.96 (1.07,3.59) |
| Tanga | 1.38 (0.77,2.45) |
| Morogoro | 0.84 (0.46,1.56) |
| Pwani | 1.24 (0.67,2.28) |
| Dar es salaam | 0.76 (0.42,1.35) |
| Lindi | 0.8 (0.43,1.49) |
| Mtwara | 0.55 (0.29,1.05) |
| Ruvuma | 1.08 (0.6,1.96) |
| Iringa | 2.7 (1.48,4.9) |
| Mbeya | 1.15 (0.63,2.09) |
| Singida | 2.24 (1.26,3.98) |
| Tabora | 1.12 (0.64,1.96) |
| Rukwa | 0.68 (0.37,1.26) |
| Kigoma | 1.13 (0.64,2) |
| Shinyanga | 0.64 (0.36,1.16) |
| Kagera | 1.95 (1.09,3.48) |
| Mwanza | 1.34 (0.76,2.35) |
| Mara | 1.06 (0.59,1.9) |
| Manyara | 1.54 (0.85,2.77) |
| Njombe | 0.6 (0.31,1.15) |
| Katavi | 1.16 (0.64,2.08) |
| Simiyu | 1.55 (0.9,2.67) |
| Geita | 1.64 (0.94,2.85) |
| Number of children under 5 | 0.91 (0.86,0.96) |
| Sex of child (REF: male) |  |
| female | 0.85 (0.75,0.96) |
| Age of child | 0.38 (0.16,0.88) |
| Age squared | 1.01 (0.99,1.04) |
| Age of head of household (REF: >50 years old) |  |
| <30 years | 0.15 (0.10,0.21) |
| 30 to 40 years | 0.67 (0.55,0.81) |
| 41 to 50 years | 1.08 (0.93,1.25) |
| Highest education attainments of the head (REF: no education) |  |
| primary | 1.68 (1.4,2.02) |
| secondary | 2.12 (1.63,2.76) |
| Travel time to nearest secondary school (<30 min) |  |
| Between 30min and 1hr | 0.89 (0.71,1.11) |
| Between 1hr and 2 hr | 1.04 (0.8,1.36) |
| More than 2hr | 0.68 (0.48,0.96) |
| Pupil to qualified teacher ratio (PQTR) | 1.01 (1,1.03) |
|  |  |
|  |  |
| Random effects | *Variance (95% CI)* |
| DHS clusters | 0.277 (0.19,0.41) |

^^^Zanzibar and islands not included because of missing data for travel time.

^*^Statistically significant at 95% confidence interval (p value < 0.05)

^**^ Statistically significant at 90% confidence interval (p value < 0.1)

^†^Small sample size, N=26
