# Supplementary material for: Understanding factors associated with attending secondary school in Tanzania using household survey data
Source: PLoS One. 2022 Feb 25;17(2):e0263734. doi: 10.1371/journal.pone.0263734 (PMC8880958; doi:10.1371/journal.pone.0263734)
Supplement: S6 Table — Full results and additional statistics. (DOCX) [file pone.0263734.s012.docx]

# SI.7.1 Table: Multilevel-multivariate analysis of drivers of school attendance, using mother and father’s information and excluding information about household head for Tanzania (2015-16). Full results and additional statistics.

Odds ratios, standard errors, z-test statistic, p-values and 95% confidence intervals from the two-level logistic regression model of school attendance among children of adolescents or youth-level using DHS data in Tanzania (2015-16 DHS, N= 6,197).

| \|  \| **Odds Ratio** \| **Std. Err.** \| **z** \| **P>z** \| **95% CI -** \| **95% CI +** \| \| --- \| --- \| --- \| --- \| --- \| --- \| --- \| \|  \|  \|  \|  \|  \|  \|  \| \| **Place of residence (REF: Urban)** \|  \|  \|  \|  \|  \|  \| \| Rural \| 0.8631412 \| 0.1129772 \| -1.12 \| 0.261 \| 0.66783 \| 1.11557 \| \|  \|  \|  \|  \|  \|  \|  \| \| **DHS Region (Dodoma)** \|  \|  \|  \|  \|  \|  \| \| Arusha \| 1.047745 \| 0.3317718 \| 0.15 \| 0.883 \| 0.56327 \| 1.94891 \| \| Kilimanjaro \| 1.908012 \| 0.5951058 \| 2.07 \| 0.038 \| 1.03536 \| 3.51617 \| \| Tanga \| 1.18471 \| 0.352097 \| 0.57 \| 0.568 \| 0.66166 \| 2.12124 \| \| Morogoro \| 0.8296214 \| 0.2631272 \| -0.59 \| 0.556 \| 0.44556 \| 1.54473 \| \| Pwani \| 1.168003 \| 0.3672531 \| 0.49 \| 0.621 \| 0.63067 \| 2.16315 \| \| Dar es salaam \| 0.7570412 \| 0.2286436 \| -0.92 \| 0.357 \| 0.41883 \| 1.36836 \| \| Lindi \| 0.7270527 \| 0.2321911 \| -1 \| 0.318 \| 0.3888 \| 1.35958 \| \| Mtwara \| 0.5131581 \| 0.1710257 \| -2 \| 0.045 \| 0.26703 \| 0.98614 \| \| Ruvuma \| 0.9222295 \| 0.2828428 \| -0.26 \| 0.792 \| 0.50557 \| 1.68228 \| \| Iringa \| 2.382236 \| 0.7334381 \| 2.82 \| 0.005 \| 1.30292 \| 4.35563 \| \| Mbeya \| 1.122678 \| 0.3463766 \| 0.38 \| 0.708 \| 0.61325 \| 2.05529 \| \| Singida \| 1.931312 \| 0.5734268 \| 2.22 \| 0.027 \| 1.07925 \| 3.45607 \| \| Tabora \| 1.041991 \| 0.3012728 \| 0.14 \| 0.887 \| 0.59123 \| 1.83643 \| \| Rukwa \| 0.5698881 \| 0.1809235 \| -1.77 \| 0.077 \| 0.30588 \| 1.06175 \| \| Kigoma \| 1.044883 \| 0.3087782 \| 0.15 \| 0.882 \| 0.5855 \| 1.86471 \| \| Shinyanga \| 0.6218613 \| 0.1891773 \| -1.56 \| 0.118 \| 0.34257 \| 1.12886 \| \| Kagera \| 1.767862 \| 0.5293788 \| 1.9 \| 0.057 \| 0.98301 \| 3.17934 \| \| Mwanza \| 1.410602 \| 0.4114804 \| 1.18 \| 0.238 \| 0.79635 \| 2.49865 \| \| Mara \| 1.080647 \| 0.3253317 \| 0.26 \| 0.797 \| 0.599 \| 1.94958 \| \| Manyara \| 1.531764 \| 0.4646992 \| 1.41 \| 0.16 \| 0.8452 \| 2.77604 \| \| Njombe \| 0.4990249 \| 0.1683987 \| -2.06 \| 0.039 \| 0.25756 \| 0.96686 \| \| Katavi \| 1.028057 \| 0.3114734 \| 0.09 \| 0.927 \| 0.56771 \| 1.86169 \| \| Simiyu \| 1.593942 \| 0.4495944 \| 1.65 \| 0.098 \| 0.91702 \| 2.77055 \| \| Geita \| 1.594726 \| 0.4548203 \| 1.64 \| 0.102 \| 0.91185 \| 2.78902 \| \|  \|  \|  \|  \|  \|  \|  \| \| **Household wealth index (Poorest)** \|  \|  \|  \|  \|  \|  \| \| Poorer \| 1.411144 \| 0.1601476 \| 3.03 \| 0.002 \| 1.12972 \| 1.76268 \| \| Middle \| 1.753441 \| 0.2037188 \| 4.83 \| < 0.001 \| 1.39636 \| 2.20184 \| \| Richer \| 3.375634 \| 0.4251141 \| 9.66 \| < 0.001 \| 2.63729 \| 4.32068 \| \| Richest \| 4.026161 \| 0.6145836 \| 9.12 \| < 0.001 \| 2.98509 \| 5.43031 \| \|  \|  \|  \|  \|  \|  \|  \| \| **Father's highest educational attainment (REF: no education)** \|  \|  \|  \|  \|  \|  \| \| Primary \| 1.979612 \| 0.3160021 \| 4.28 \| < 0.001 \| 1.44779 \| 2.7068 \| \| Secondary \| 4.65982 \| 1.406448 \| 5.1 \| < 0.001 \| 2.57903 \| 8.41943 \| \| Higher \| 5.57468 \| 4.631638 \| 2.07 \| 0.039 \| 1.09399 \| 28.4072 \| \| not present in hh \| 1.250286 \| 0.2034897 \| 1.37 \| 0.17 \| 0.90881 \| 1.72007 \| \|  \|  \|  \|  \|  \|  \|  \| \| **Mother's highest educational attainment (REF: no education)** \|  \|  \|  \|  \|  \|  \| \| Primary \| 1.452634 \| 0.1574854 \| 3.44 \| 0.001 \| 1.17456 \| 1.79655 \| \| Secondary \| 4.255325 \| 1.333727 \| 4.62 \| < 0.001 \| 2.30221 \| 7.8654 \| \| Higher \| 1.192062 \| 1.610042 \| 0.13 \| 0.897 \| 0.08446 \| 16.8253 \| \| not present in hh \| 0.6723575 \| 0.0775923 \| -3.44 \| 0.001 \| 0.53625 \| 0.84301 \| \|  \|  \|  \|  \|  \|  \|  \| \| **Sex of child (Male)** \|  \|  \|  \|  \|  \|  \| \| female \| 0.8468451 \| 0.0542176 \| -2.6 \| 0.009 \| 0.74698 \| 0.96006 \| \| **Number of children under the age of 5** \| 0.9101221 \| 0.0260296 \| -3.29 \| 0.001 \| 0.86051 \| 0.9626 \| \| **Age of child** \| 0.2662654 \| 0.1151162 \| -3.06 \| 0.002 \| 0.11411 \| 0.62133 \| \| **Age squared** \| 1.027984 \| 0.0136285 \| 2.08 \| 0.037 \| 1.00162 \| 1.05505 \| \|  \|  \|  \|  \|  \|  \|  \| \| **Travel time to nearest secondary school (<30 min)** \|  \|  \|  \|  \|  \|  \| \| Between 30min and 1hr \| 0.9149642 \| 0.1073216 \| -0.76 \| 0.449 \| 0.72705 \| 1.15145 \| \| Between 1hr and 2 hr \| 1.035984 \| 0.1423867 \| 0.26 \| 0.797 \| 0.79134 \| 1.35626 \| \| More than 2hr \| 0.6702786 \| 0.1209779 \| -2.22 \| 0.027 \| 0.47057 \| 0.95475 \| \|  \|  \|  \|  \|  \|  \|  \| \| **Pupil to qualified teacher ratio (PQTR)** \| 1.014303 \| 0.0077998 \| 1.85 \| 0.065 \| 0.99913 \| 1.02971 \| \| _cons \| 229125 \| 805179.9 \| 3.51 \| < 0.001 \| 233.787 \| 2.25E+08 \| \|  \|  \|  \|  \|  \|  \|  \| \|  \|  \|  \|  \|  \|  \|  \| \| **Random-effects Parameters** \| **Estimate** \| **Std. Err.** \|  \|  \| **95% CI -** \| **95% CI +** \| \|  \|  \|  \|  \|  \|  \|  \| \| **DHS clusters** \| 0.2865643 \| 0.0553539 \|  \|  \| 0.19625 \| 0.41845 \| |
| --- | --- | --- | --- | --- | --- | --- | --- | --- | --- | --- | --- | --- | --- | --- | --- | --- | --- | --- | --- | --- | --- | --- | --- | --- | --- | --- | --- | --- | --- | --- | --- | --- | --- | --- | --- | --- | --- | --- | --- | --- | --- | --- | --- | --- | --- | --- | --- | --- | --- | --- | --- | --- | --- | --- | --- | --- | --- | --- | --- | --- | --- | --- | --- | --- | --- | --- | --- | --- | --- | --- | --- | --- | --- | --- | --- | --- | --- | --- | --- | --- | --- | --- | --- | --- | --- | --- | --- | --- | --- | --- | --- | --- | --- | --- | --- | --- | --- | --- | --- | --- | --- | --- | --- | --- | --- | --- | --- | --- | --- | --- | --- | --- | --- | --- | --- | --- | --- | --- | --- | --- | --- | --- | --- | --- | --- | --- | --- | --- | --- | --- | --- | --- | --- | --- | --- | --- | --- | --- | --- | --- | --- | --- | --- | --- | --- | --- | --- | --- | --- | --- | --- | --- | --- | --- | --- | --- | --- | --- | --- | --- | --- | --- | --- | --- | --- | --- | --- | --- | --- | --- | --- | --- | --- | --- | --- | --- | --- | --- | --- | --- | --- | --- | --- | --- | --- | --- | --- | --- | --- | --- | --- | --- | --- | --- | --- | --- | --- | --- | --- | --- | --- | --- | --- | --- | --- | --- | --- | --- | --- | --- | --- | --- | --- | --- | --- | --- | --- | --- | --- | --- | --- | --- | --- | --- | --- | --- | --- | --- | --- | --- | --- | --- | --- | --- | --- | --- | --- | --- | --- | --- | --- | --- | --- | --- | --- | --- | --- | --- | --- | --- | --- | --- | --- | --- | --- | --- | --- | --- | --- | --- | --- | --- | --- | --- | --- | --- | --- | --- | --- | --- | --- | --- | --- | --- | --- | --- | --- | --- | --- | --- | --- | --- | --- | --- | --- | --- | --- | --- | --- | --- | --- | --- | --- | --- | --- | --- | --- | --- | --- | --- | --- | --- | --- | --- | --- | --- | --- | --- | --- | --- | --- | --- | --- | --- | --- | --- | --- | --- | --- | --- | --- | --- | --- | --- | --- | --- | --- | --- | --- | --- | --- | --- | --- | --- | --- | --- | --- | --- | --- | --- | --- | --- | --- | --- | --- | --- | --- | --- | --- | --- | --- | --- | --- | --- | --- | --- | --- | --- | --- | --- | --- | --- | --- | --- | --- | --- | --- | --- | --- | --- | --- | --- | --- | --- | --- | --- | --- | --- | --- | --- | --- | --- | --- | --- | --- | --- | --- | --- | --- | --- | --- | --- | --- | --- | --- | --- | --- | --- | --- | --- | --- | --- | --- | --- | --- | --- | --- | --- | --- | --- | --- | --- | --- | --- | --- | --- | --- | --- | --- | --- | --- | --- | --- | --- | --- | --- | --- | --- | --- | --- | --- | --- | --- | --- | --- | --- | --- | --- | --- | --- | --- | --- | --- | --- | --- | --- | --- | --- | --- | --- | --- | --- | --- | --- | --- | --- | --- | --- | --- | --- | --- | --- | --- | --- | --- | --- | --- | --- | --- |
