# Supplementary material for: Understanding factors associated with attending secondary school in Tanzania using household survey data
Source: PLoS One. 2022 Feb 25;17(2):e0263734. doi: 10.1371/journal.pone.0263734 (PMC8880958; doi:10.1371/journal.pone.0263734)
Supplement: S7 Table — (DOCX) [file pone.0263734.s013.docx]

# SI.7 Table: Multilevel-multivariate analysis of drivers of school attendance,

# using mother and father’s information and excluding information about

# household head for Tanzania (2015-16).

Odds ratios and 95% confidence intervals from the two-level logistic regression model of school attendance among children of adolescents or youth-level using DHS data in Tanzania (2015-16 DHS, N= 6,197).

| **Fixed effects** | Odds Ratios (95% CI) | |
| --- | --- | --- |
| **Place of residence (REF: Urban)** |  | |
| Rural | 0.86 (0.67,1.12) | |
| **DHS Region (Dodoma)** |  | |
| Arusha | 1.05 (0.56,1.95) | |
| Kilimanjaro | 1.91 (1.04,3.52)* | |
| Tanga | 1.18 (0.66,2.12) | |
| Morogoro | 0.83 (0.45,1.54) | |
| Pwani | 1.17 (0.63,2.16) | |
| Dar es salaam | 0.76 (0.42,1.37) | |
| Lindi | 0.73 (0.39,1.36) | |
| Mtwara | 0.51 (0.27,0.99)* | |
| Ruvuma | 0.92 (0.51,1.68) | |
| Iringa | 2.38 (1.3,4.36)* | |
| Mbeya | 1.12 (0.61,2.06) | |
| Singida | 1.93 (1.08,3.46)* | |
| Tabora | 1.04 (0.59,1.84) | |
| Rukwa | 0.57 (0.31,1.06) | |
| Kigoma | 1.04 (0.59,1.86) | |
| Shinyanga | 0.62 (0.34,1.13) | |
| Kagera | 1.77 (0.98,3.18) | |
| Mwanza | 1.41 (0.8,2.5) | |
| Mara | 1.08 (0.6,1.95) | |
| Manyara | 1.53 (0.85,2.78) | |
| Njombe | 0.5 (0.26,0.97)* | |
| Katavi | 1.03 (0.57,1.86) | |
| Simiyu | 1.59 (0.92,2.77) | |
| Geita | 1.59 (0.91,2.79) | |
| **Household wealth index (Poorest)** |  | |
| Poorer | 1.41 (1.13,1.76)* | |
| Middle | 1.75 (1.4,2.2)* | |
| Richer | 3.38 (2.64,4.32)* | |
| Richest | 4.03 (2.99,5.43)* | |
| **Father's highest educational attainment (REF: no education)** †† |  | |
| Primary | 1.98 (1.45,2.71)* | |
| Secondary | 4.66 (2.58,8.42)* | |
| Higher | 5.57 (1.09,28.41)* | |
| not present in hh | 1.25 (0.91,1.72) | |
| **Mother's highest educational attainment (REF: no education)** † |  | |
| Primary | 1.45 (1.17,1.8)* | |
| Secondary | 4.26 (2.3,7.87)* | |
| Higher | 1.19 (0.08,16.83) | |
| not present in hh | 0.67 (0.54,0.84)* | |
| **Sex of child (Male)** |  | |
| female | 0.85 (0.75,0.96)* | |
| **Number of children under the age of 5** | 0.91 (0.86,0.96)* | |
| **Age of child** | 0.27 (0.11,0.62)* | |
| **Age squared** | 1.03 (1,1.06) | |
| **Travel time to nearest secondary school (<30 min)** |  | |
| Between 30min and 1hr | 0.91 (0.73,1.15) | |
| Between 1hr and 2 hr | 1.04 (0.79,1.36) | |
| More than 2hr | 0.67 (0.47,0.95)* | |
| **Pupil to qualified teacher ratio (PQTR)** | 1.01 (1,1.03)* | |
|  |  | |
| **Random effects** | Variance (95% CI) | |
|  |  | |
| DHS clusters | 0.286 (0.20,0.42)* | |
| ^Zanzibar and islands not included because of missing data for travel time.  *Statistically significant at 95% confidence interval (p value < 0.05)  †Total number of valid observations available for mothers' sample N=3,589 (mothers present in the hh)  ††Total number of valid observations available for father' sample N=2,288 (fathers present in the hh) | |  |
