# Supplementary material for: Understanding factors associated with attending secondary school in Tanzania using household survey data
Source: PLoS One. 2022 Feb 25;17(2):e0263734. doi: 10.1371/journal.pone.0263734 (PMC8880958; doi:10.1371/journal.pone.0263734)
Supplement: S8 Table — Tanzania DHS2015-16. (DOCX) [file pone.0263734.s014.docx]

# SI.10 Table: Likelihood ratio tests for null against full models. Tanzania

# DHS2015-16.

Akaike’s information criterion and Bayesian information criterion

| Model | Obs | ll (null) | ll (model) | df | AIC | BIC |
| --- | --- | --- | --- | --- | --- | --- |
| Null model | 6,197 | . | -3952.995 | 2 | 7909.991 | 7923.455 |
| Full model | 6,197 | . | -3285.795 | 46 | 6663.59 | 6973.254 |

Likelihood ratio test: LR chi2 (44) = 1334.40 (assumption: null nested in full model)
