# Supplementary material for: Understanding factors associated with attending secondary school in Tanzania using household survey data
Source: PLoS One. 2022 Feb 25;17(2):e0263734. doi: 10.1371/journal.pone.0263734 (PMC8880958; doi:10.1371/journal.pone.0263734)
Supplement: S1 Text — (DOCX) [file pone.0263734.s015.docx]

# SI.1 Text: Distribution of the pupil-qualified teacher ratio (PQTR) in Tanzania.

Map **A)** shows the ratios at school level, where green indicates a low ratio and red indicates a high ratio. Source of school level data: Tanzania Opendata - Consolidated Secondary School Data by Age and Sex – 2018 [46]. **B)** shows the interpolated surface of the PQTR realised using inverse distance weighting on same colour scheme.

Figure 1a shows the distribution of the PQTR in Tanzania. The mean PQTR was 21.0 pupils per teacher and the median 19.8 (standard deviation 9.13). Please note that this figure differs slightly from the mean PQTR cluster level figure, as the latter was calculated after the cluster level extraction. Figure 1b shows the gridded PQTR based on IDW. The map provides a proxy estimate education quality across Tanzania. Note that, population density or the absolute number of children were not taken into account in the maps. Thus, areas with better estimate of PQTR may be sparsely populated.

Inverse Distance Weighting (IDW) interpolation technique exploits information from known data points to estimate other values at unknown locations, where observed values are not available, to create a continuous raster surface of a certain indicator. The assumption behind IDW is that the influence of each known point to the surrounding areas decreases with distance; therefore, when creating the surface, IDW assigns greater weights to the known points closer to the area to be estimated, and lower weights to the points which are farther away. The area to be estimated therefore is calculated as a weighted average of surrounding known values [68-70].

**References**68. Shepard D. A two-dimensional interpolation function for irregularly-spaced data. Proceedings. 1968;23rd ACM National Conference. ACM’68; New York, NY: ACM; 1968. pp. 517–524.

69. Adebayo O, Abimbola S, Kolawole O, Taiwo O. Mapping of river waterquality using inverse distance weighted interpolation in Ogun-Osun river basin, Nigeria. Acta Geographica Debrecina Landscape &amp; Environment. 2013;7(2).

70. ESRI. How IDW works 2019 [Available from: <http://desktop.arcgis.com/en/arcmap/latest/tools/spatial-analyst-toolbox/how-idw-works.htm>
