# Supplementary material for: Understanding factors associated with attending secondary school in Tanzania using household survey data
Source: PLoS One. 2022 Feb 25;17(2):e0263734. doi: 10.1371/journal.pone.0263734 (PMC8880958; doi:10.1371/journal.pone.0263734)
Supplement: S2 Text — (DOCX) [file pone.0263734.s016.docx]

# SI.11 Text: QQplot: check for normality of residuals assumption for the main household models.

Overall, there is some deviation from the expected normal line towards the tails, however the line looks straight and therefore pretty normal and suggests that the normality assumption for level 2 (cluster) residuals is not violated.
