# Supplementary material for: Understanding factors associated with attending secondary school in Tanzania using household survey data
Source: PLoS One. 2022 Feb 25;17(2):e0263734. doi: 10.1371/journal.pone.0263734 (PMC8880958; doi:10.1371/journal.pone.0263734)
Supplement: S3 Text — (DOCX) [file pone.0263734.s017.docx]

SI.12 Text: Calibration of the model's predictions plot for goodness of fit of the model.

Observed versus predicted probabilities using 10 bins. In Tanzania, scatter points are near the diagonal line in the graph therefore we have good fit of the model.
